# Comparative meta-RNA-seq of the vaginal microbiota and differential expression by *Lactobacillus iners* in health and dysbiosis

**DOI:** 10.1186/2049-2618-1-12

**Published:** 2013-04-12

**Authors:** Jean M Macklaim, Andrew D Fernandes, Julia M Di Bella, Jo-Anne Hammond, Gregor Reid, Gregory B Gloor

**Affiliations:** 1 Department of Biochemistry, The University of Western Ontario, London N6A 5C1, Canada; 2 Canadian Research & Development Centre for Probiotics, Lawson Health Research Institute, London, ON, N6A 4V2, Canada; 3 YouKaryote Genomics, 2036 Riverbend Road, London, ON, N6K 0A1, Canada; 4 Department of Microbiology and Immunology, The University of Western Ontario, London, N6A 5C1, Canada; 5 Department of Family Medicine, The University of Western Ontario, London, N6A 5C1, Canada; 6 Department of Surgery, The University of Western Ontario, London, N6A 5C1, Canada

**Keywords:** Bacterial vaginosis, Vaginal microbiome, Meta-transcriptomics, High-throughput sequencing, RNAseq

## Abstract

**Background:**

Bacterial vaginosis (BV), the most common vaginal condition of reproductive-aged women, is associated with a highly diverse and heterogeneous microbiota. Here we present a proof-of-principle analysis to uncover the function of the microbiota using meta-RNA-seq to uncover genes and pathways that potentially differentiate healthy vaginal microbial communities from those in the dysbiotic state of bacterial vaginosis (BV).

**Results:**

The predominant organism, *Lactobacillus iners*, was present in both conditions and showed a differing expression profile in BV compared to healthy. Despite its minimal genome, *L. iners* differentially expressed over 10% of its gene complement. Notably, in a BV environment *L. iners* increased expression of a cholesterol-dependent cytolysin, and of mucin and glycerol transport and related metabolic enzymes. Genes belonging to a CRISPR system were greatly upregulated suggesting that bacteriophage influence the community. Reflective of *L. iners*, the bacterial community as a whole demonstrated a preference for glycogen and glycerol as carbon sources under BV conditions. The predicted end-products of metabolism under BV conditions include an abundance of succinate and other short-chain fatty-acids, while healthy conditions are predicted to largely contain lactic acid.

**Conclusions:**

Our study underscores the importance of understanding the functional activity of the bacterial community in addition to characterizing the population structure when investigating the human microbiome.

## Background

Studies using high-throughput metagenomic and 16S rRNA sequencing have identified over 250 bacterial types in the human vagina. Microbial profiles in women who are clinically healthy most often have a low diversity microbiota dominated by lactobacilli with the most common species being *Lactobacillus crispatus*, *Lactobacillus iners*, *Lactobacillus jensenii*, and *Lactobacillus gasseri*[1-4]. Conversely, for bacterial vaginosis (BV), an aberrant condition associated with increased risk of sexually transmitted infections and preterm labor [5,6], several high-diversity, multi-species profiles have been reported [3,7]. This makes the human vaginal microbiota different than other human microbial ecosystems where a high species diversity correlates with healthy conditions. For example and in contrast, inflammatory bowel disease is characterized by a loss of diversity [8]. The vaginal microbiota is highly dynamic and bacterial populations can change rapidly between the healthy and BV states [1,4,9], but the cause for these transitions is unknown. The most frequently detected organism, *Lactobacillus iners*, appears to have a streamlined genome adapted for persistence in the vagina [10]. This organism is detected in women regardless of BV status [7,11], but not much is known about how *L. iners* can adapt to these differing environments or its role in the etiology and pathogenesis of BV.

Although there are numerous 16S rRNA studies of the vaginal microbiome that analyze the relative microbial composition, for example, Ravel *et al*., Hummelen *et al*., and Lamont *et al*. [1,3,12], none have yet attempted to characterize the function of the microbiota using culture-independent methods. We therefore chose a meta-transcriptomic approach using RNA-seq to address the functional contribution of the bacterial community. We further sought to understand the function of *Lactobacillus iners*, an organism found nearly ubiquitously in the vagina.

## Results and discussion

Vaginal swabs from four women were collected for RNA-seq of the bacterial transcriptome. Two women were diagnosed with BV and two with a healthy (non-BV, N) vaginal profile according to the low Nugent score, vaginal *p*H, and signs and symptoms of the condition as noted by the examining clinician (see Additional file 1: Table S1 for Nugent scoring). Sequenced reads were mapped against a reference coding sequence library (refseqs - see Methods) resulting in 5,487,128 to 10,635,713 uniquely mapped reads per sample, far exceeding the sequencing depth of recent RNA-seq studies [13-15]. The number of unique refseqs mapped ranged from 10,770 to 22,860 per sample, with more refseqs expectantly identified in BV samples due to the higher microbial diversity (mapping is summarized in Additional file 1: Table S2). Among all four samples, 33,412 unique refseqs were identified in total. To verify the completeness of our reference library and ensure no major known taxon was missing from analysis, we performed an independent mapping against a reference *cpn60* gene database. The highly expressed *cpn*60 (or *groEL*) gene is nearly universally conserved in bacteria and contains a variable sequence, which allows for taxonomic discrimination [16], and unlike the rRNA molecules, is not depleted by the mRNA enrichment process. Mapped *cpn60* reads representing at least 0.1% of the total *cpn60* mapped reads are presented in Additional file 1: Table S3. The data suggested that our reference library contained representative genomes for the most abundant organisms detected in our samples, and that any unavailable genomes would not make up an appreciable fraction of the bacterial or mRNA population.

**Figure 1:**
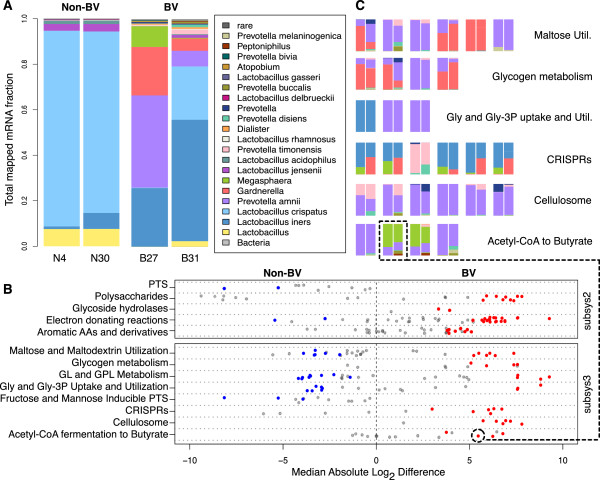
**Bacterial community structure and function. **(**A**) The distribution of all mapped mRNA reads for each taxon per sample. The two samples that were considered healthy (N4 and N30) are dominated by Lactobacillus crispatus with lesser amounts of L. iners and L. jensenii detected. The two samples having bacterial vaginosis (BV) have a lower relative proportion of lactobacilli and large proportions of Prevotella amnii, Gardnerella vaginalis, Megasphaera, and other anaerobic organisms. (**B**) Selected subsystem 2 and subsystem 3 categories showing enriched subsystem 4 functions under N or BV conditions. Each point represents a specific subsystem 4 function, and the magnitude of change in expression is plotted on the x-axis relative to the BV condition. Points are colored blue (non-BV) or red (BV) if significantly differentially expressed between conditions, whereas grey points are not differential between the conditions. (**C**) Representation of the fractional taxon contribution to functions significantly upregulated in BV (red points in B). Pairs of bars represent the two samples (B27 and B31) and each color is the fraction of reads for that function contributed by a particular taxon. Butyrate kinase, as part of the Acetyl-CoA fermentation to Butyrate subsystem, is highlighted to show the relationship between the differential functions in 1B and the taxonomic composition of the function in 1C. Gly, Glycerol; Gly-3P, Glycerol-3-phosphate; GL, Glycerolipid; GPL, Glycerophospholipid.

The fraction of mapped reads per taxon for each sample is represented in Figure 1A. The microbiota associated with health, samples N4 and N30, were dominated by lactobacilli (*L. iners* and *L. crispatus*), while those associated with BV, B27 and B31, were a mixed population composed of lactobacilli and anaerobic organisms that included *Prevotella*, *Gardnerella,* and *Megasphaera*. The profiles are consistent with those from healthy women and those with BV studied by others [1-4], and corresponded to the Nugent profile for each sample (Additional file 1: Table S1).

The multi-organism composition of our samples and the resulting gene diversity violates assumptions of current differential RNA-seq analysis methods. These methods generally perform well when used to study the transcriptome of single organisms, however they implicitly assume that the expected between-condition difference is essentially a fixed effect [17,18]. Since each sample in this study was obtained from a different subject, a random-effect type model is more appropriate. However, it can be problematic to use this model when there are not enough samples to accurately estimate the additional parameter(s) required by random-effect models. We therefore developed a specialized statistical framework termed analysis of variance (ANOVA)-like differential expression (ALDEx) (Fernandes *et al*., unpublished work) that infers differential expression by estimating the magnitude of between-condition expression difference with respect to within-condition expression difference. Thus genes (or functional groups) identified as differentially expressed between the N and BV conditions have a between-condition (N vs BV) expression difference that is large in comparison to the within-condition (B27 + B31 and N4 + N30) variability. Furthermore, since we posit that biochemical function, not organism is the major unit of analysis for the vaginal microbiome, the data were grouped via refseqs into functional groups (KO or SEED subsys4) unless otherwise noted. Functional-level analyses of metagenomic data, as opposed to gene-level analyses, have been used previously [19]. Unsupervised hierarchical clustering showed that samples were more similar when refseqs were grouped by function (Additional file 1: Figure S2B) than clustering by refseq expression (Additional file 1: Figure S2A). We also noted, regardless of refseq grouping, the two BV samples were most similar to each other based on gene expression and were separable from the two N samples that also clustered together. Estimated between- vs within-expression ratios along with read counts and annotations are presented in Additional file 2: Table S8 (*iners* refseqs), Additional file 3: Table S9 (subsys4) and Additional file 4: Table S10 (KEGG KOs).

### Response of *L. iners *to BV

*L.iners *is the most frequently detected vaginal organism, and is known for its consistent presence in N and BV states [1,3,20]. We detected *L. iners* in our samples with an average coding sequence (CDS) coverage per sample ranging from approximately 5-fold to 117-fold (Additional file 1: Table S5). This allowed us to uncover gene expression differences in this organism between the two vaginal conditions.

**Figure 2:**
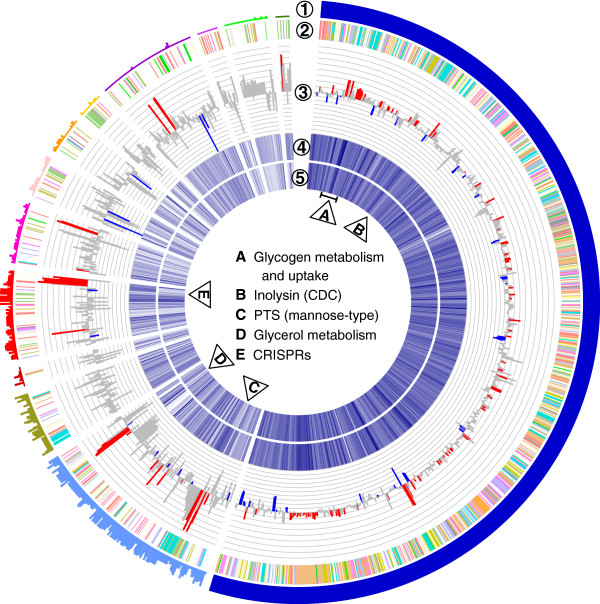
**Circular representation of RNA-seq data for the Lactobacillus iners pan-transcriptome.** RNA-seq reads were mapped to 12 available L. iners genomes (listed in Additional file 1: Table S4) after clustering redundant coding sequences by nucleotide identity (see Methods). Breaks in the circle separate blocks of contiguous coding sequences (CDS) ordered by scaffolding on the genome assemblies (Ring 1). The height of the plot represents in how many of the 12 genomes the CDS occurs. The first contiguous block (dark blue) are the 954 CDS present in all genomes and are considered the core L. iners gene set. Ring 2 shows the clusters of orthologous groups (COG) color-coded function of each CDS. Ring 3 The differential expression of each CDS between non-bacterial vaginosis (BV) and BV samples. The absolute height of the bar shows the fold-change (log2) in expression (positive for up BV, and negative for up non-BV). Bars are colored if significantly differential (red for BV and blue for non-BV). Regions of interest are labeled in the centre of the circle. Rings 4, and 5, show a heatmap representation of the median CDS expression in BV and non-BV samples, respectively. Darker blue represents higher expression. Regions of interest noted in the results are marked by lettered triangles.

We mapped a total of 1,653 unique *L. iners* CDS with at least one read and refer to this set as the *L. iners* pan-transcriptome (Figure 2). There were 954 of the 1,653 CDS present in all 12 *L. iners* genomes that represent the core-expressed CDS set. The core functions, in general, had a high relative expression and low fold-change between conditions (Figure 2). A total of 207 CDS were inferred to be differentially regulated due to their large estimated between- vs -within expression ratios. These ratios, along with read counts and annotations, are presented in Additional file 2: Table S8.

Amongst the most highly differentiated CDS were eight contiguous genes encoding the proteins for the CRISPR anti-bacteriophage defense system and include the 5-member (CasA-CasE) protein complex, Cascade (CRISPR-associated complex for antiviral defense) [21]. These CRISPR-related genes were highly expressed only under BV conditions (Figure 1B, C, and Figure 2) suggesting that *L. iners* is responding to an altered environmental phage load. Interestingly, the cas proteins were only present in 4/12 *L. iners* genomes (Figure 2) which raises the question of strain-specific adaptations to the vaginal environment that could not be identified by 16S rRNA profiling. It is also possible that these differences reflect different genome assembly qualities. We noted that restriction-modification systems were also highly upregulated by *L. iners* in BV (Additional file 1: Figure S3), perhaps as further defense against bacteriophage infection.

The cas proteins, as part of the CRISPR anti-bacteriophage system, regulate the insertion and presentation of small pieces of DNA spacer sequences in the bacterial chromosome used to inactivate attacking phage DNA [22]. We therefore probed our RNA-seq data for evidence of CRISPR spacer sequences and detected between 21,477 and 225,242 potential CRISPR spacers per sample of which the vast majority corresponded to known sequences of *Lactobacillus* origin (Additional file 1: Table S6). Examining the meta-RNAseq data also revealed six highly upregulated CRISPR-associated proteins (Figure 1B). Most of the reads were contributed by *L. iners* (as described above), but there was shared expression of these functions by *G. vaginalis* and *Megasphaera*, and one of the cas proteins was expressed only by *Prevotella* species (Figure 1C). Our observations are supported by recent evidence that several *Lactobacillus*-specific bacteriophage have been isolated from women in South Africa with and without BV [23], and that metagenomic data from the Human Microbiome Project has also revealed a diversity of CRISPRs in all body sites, with an abundance of *Lactobacillus*-targeting phage sequence in the vagina [24].

A notable core CDS, a cholesterol-dependent cytolysin (CDC) encoded by *L. iners*[10], was upregulated at least 6-fold in BV (Figure 2B). This CDC is active between *p*H 4.5 and 6.0 [25], which corresponds to the elevated *p*H range of BV [26]. The predicted protein is similar to vaginolysin, another CDC present in *G. vaginalis* that is upregulated at least 256-fold in the meta-RNA-seq analysis (Additional file 3: Table S9). Vaginolysin has been shown to have cytotoxic activity towards human erythrocytes, vaginal epithelial, and cervical cells [27]. This raises the possibility that the *L. iners*-encoded CDC may play an unappreciated role in BV and might contribute to the pathogenesis of the condition.

Examination of the distribution of expressed reads assigned to clusters of orthologous groups (COG) functions showed an increase in transcriptional effort by *L. iners* for carbohydrate transport and metabolism and a number of individual CDS under this category were upregulated in BV (Additional file 1: Figure S4). In the genomic context, three separate loci involved in carbohydrate uptake were upregulated 3- to 9-fold in BV (Figure 2: two loci are marked in the region labeled A and the third in the region labelled C). The first is a broad-specificity phosphoenolpyruvate-dependent transport system (PTS) of the mannose family along with a putative regulator of the mannose operon, ManO, possibly targeting carbohydrate moieties of the vaginal mucosa [10]. This PTS is conserved in all 12 genomes of *L. iners* suggesting its functional importance to the organism in the vagina. A second region, also conserved across all sequenced *L. iners* genomes, contains an ABC-type maltose transport system next to a maltose phosphorylase suggesting a preference for maltose uptake under BV. The source of maltose could be from breakdown of glycogen by *L. iners*, and consistent with this, there are four upregulated glycosylases predicted to target α-1,6-glucocidic linkages that bridge the branching points in glycogen. The third locus related to carbohydrate utilization contains a second mannose-family PTS flanked by an oligo-1,6-glucosidase de-branching enzyme and a LacI family transcriptional regulator. This region is present in only six of the twelve available *L. iners* genomes and suggests that only a subset of strains are able to use these genes for adaptation during BV.

Though glycogen is thought to be the major carbon source for vaginal bacteria [28], we surprisingly noted under BV conditions the upregulation of a tandem set of three genes related to glycerol metabolism, including a glycerol kinase, a glycerol-3-phosphate dehydrogenase, and a glycerol facilitator (glpF) (region marked D in Figure 2). The genes together suggest that *L. iners* is able to uptake glycerol for conversion to glycerol-3-phosphate and then glycerone phosphate for entry into glycolysis or glycerophospholipid metabolism.

We examined the glycerol utilization genes in the context of the meta-transcriptome (Figure 1B and C). The glpF glycerol transporter and downstream glycerol kinase are highly expressed by both *L. iners* and *L. crispatus* and therefore results in no differential expression of these genes at the community level. In addition to the glpF transporter, *L. iners* and *L. crispatus* express a UGP-type glycerol-3P transporter more strongly under N conditions. *Prevotella amnii*, a BV-associated organism, also expressed an independent glycerol-3P uptake system (glpT) (Additional file 3: Table S9). By-passing the ATP-using phosphorylation of glycerol by glycerol kinase, glpT and ugpB/E allows direct uptake of glycerol-3-phosphate, which the organism can use for phospholipid biosynthsis or conversion to glycerone-P using glycerol-3-phosphate dehydrogenase (expressed highly in BV) for entry into glycolysis. Two enzymes (Glycerol-3-phosphate cytidylyltransferase and Glycerol-3-phosphate O-acyltransferase) are expressed highly under N conditions by *L. iners* and *L. crispatus* and indicate glycerol-3P is also converted into products for lipoteichoic acid and glycerophospholipid synthesis. The overall high expression and the differential transcription of these glycerol-related genes suggests glycerol is an underappreciated molecule in the vaginal bacterial ecosystem.

As an additional evaluation of gene expression by *L. iners*, we collected and sequenced mRNA from a single *L. iners* strain, AB-1, grown to mid-log phase in MRS broth (Difco, BD Detroit, MI, USA). Additional file 1: Figure S10 and Additional file 2: Table S8 summarize the mapping and gene expression for this sample. Similar to the *in vitro* expression data, core genes were highly expressed *in vitro* and many potentially strain-specific genes were not detected. Regions for carbohydrate uptake and metabolism (marked A, C, and D in Figure 1 and in Additional file 1: Figure S10) were amongst the most highly expressed. One notable difference between the *in vitro* expression compared to the clinical samples was the lack of CRISPR expression (region E in Figure 1A and in Additional file 1: Figure S10). Also notable, the cytolysin is highly expressed under culture conditions. These data show that genes that may be key for survival of *L. iners* in the vagina are induced under *in vitro* conditions, opening possibilities for future experiments to test gene expression and the responding phenotype.

### The vaginal community in BV

As described in Methods, we grouped refseqs (representing genes from one or multiple organisms) by their annotated function in order to evaluate functional differences between N and BV samples that may be contributed by multiple organisms. Using SEED Subsystem annotations, we estimated expression differences for subsys level 4 functions using ALDEx (output is presented in Additional file 3: Table S9). The expression of subsys4 level functions were visually represented in two ways: 1) as strip plots where the subsys4 functions were plotted by absolute fold-change between conditions (Figure 1B, and Additional file 1: Figure S5A, B and C) colored by significance and 2) as heatmaps where the summed expression value for each subsys4 was averaged over the number of genes per function (Additional file 1: Figure S6A, B and C). The strip plot allows us to visualize under which functional category the subsys4 functions are binned, while the heatmaps demonstrate the transcriptional effort by the community for each function based on the subsys4 expression relative to the average expression of all subsys4. In addition to identifying differential functions, we can show the taxonomic composition of the function (Figure 1C, and Additional file 1: Figures S7 and S8, and in Additional file 5: Table S11, Additional file 6: Table S12, Additional file 7: Table S13, Additional file 8: Table S14), allowing us to discern which organisms are producing transcripts for each function.

At the broadest functional level (SEED subsystem 1), the most highly expressed subsys4 functions in both conditions belong to general cell maintenance and information processing functions such as cell division and cell cycle, nucleosides and nucleotides, and RNA and DNA metabolism (Additional file 1: Figure S6A). However, many of the differentially expressed subsys4 functions for these categories were more highly expressed under the N condition (Additional file 1: Figure S5A). The trend was conserved at the more specific SEED subsystems level 2 and level 3 (Additional file 1: Figures S5B and C, and S6B and C). This management of cell growth and proliferation is consistent with known constraints on apportioning cellular resources [29]. We suggest that some of the transcriptional resources are necessarily diverted from housekeeping tasks to other functions in BV because of the greater diversity of organisms with a wider array of metabolic capabilities.

Similar to the findings for *L. iners*, several functions related to carbohydrate metabolism were found to differ between N and BV samples at the SEED subsystem 2 and 3 levels (summarized in Figure 1B, and Additional file 1: Figure S5B and C). The BV samples were enriched at subsystem 2 functional categories of Glycoside hydrolases, and Polysaccharides, and also at subsystem 3 in Cellulosome, and Glycogen metabolism. The functions belonging to these categories are enzymes involved in uptake, degradation, and metabolism of glycans. Considering the context of the vagina, these glycan-targeting genes are likely used for the metabolism of glycogen, which accumulates in the vaginal epithelium [30]. The genes involved in these functions largely belong to *P. amnii* and some to *G. vaginalis* (Additional file 9: Table S7). In addition to several glycosidases and glycosyltransferases, there are components of an outer membrane-bound starch utilization system (Sus), belonging to *P. amnii*, which are expressed in BV and have been implicated in binding and transporting of starch by several intestinal bacteria [31]. Interestingly, the upregulation of a starch phosphorylase gene by *P. amnii* indicates it may be able to synthesize glycogen via UDP-glucose; a phenomenon observed in other *Prevotella* isolated from the ruminal gut [32]. This could allow *Prevotella* to control carbohydrate resources by storing excess for a time when it might be needed due to growth needs, or limits in environmental carbon sources. Supporting glycogen as an important carbon source for the BV organisms is the upregulation of oligo-1,6-glucosidase and glucokinase, the enzymes responsible for directing glycogen into glycolysis via alpha-D-glucose.

Similar to the SEED subsystem analysis, we next grouped the refseqs by Kyoto Encyclopedia of Genes and Genomes (KEGG) KO numbers for differential expression analysis. We used the KO and EC assignments to place differential functions in corresponding KEGG pathways to examine the community gene functions in the context of linked metabolic pathways. Examined this way, the two conditions differed primarily in energy metabolism. The major steps of glycolysis and the conversion of pyruvate to lactic acid was upregulated under N conditions, indicating that lactic acid was the main metabolic end-product, consistent with the predominance of lactobacilli in these samples. In contrast, for the BV state, SEED subsystem 2 (Figure 1B, and Additional file 1: Figure S5B) showed a number of upregulated functions in electron-donating reactions belonging to steps of the tricarboxylic acid (TCA) cycle and oxidative phosphorylation leading to the production of succinate. A high ratio of succinate to lactic acid was the original marker of BV [33,34], and high succinate concentrations distinguish BV from the similar but distinct condition of aerobic vaginitis (AV) [35]. Increasing succinate at the expense of lactate results in a self-perpetuating condition whereby an elevated *p*H and a more reduced environment [36] supports the growth of the anaerobic bacteria noted in BV. In addition to succinate, we observed an upregulation of butyryl-CoA dehydrogenase and butyrate kinase leading to butyrate production from acetyl-CoA under BV conditions. Examination of these functions linked to butyrate production in SEED subsystems showed these were expressed largely by *P. amnii* and by *Megasphaera*. Butyryl-CoA dehydrogenase and butyrate kinase are shown as first and second enzymes in the Acetyl-CoA to Butyrate function in Figure 1C. These short-chain fatty acids have been shown to modulate immune function in the vagina and these anti-inflammatory properties may contribute to the non-inflammatory nature of BV in contrast to the inflammatory AV condition [35,37,38].

One of the distinctive characteristics of symptomatic BV is the fishy odor upon application of 10% KOH attributed to increased polyamines in the vaginal fluid produced by amino acid breakdown [39]. *P. amnii* expressed an arginine decarboxylase under BV conditions, which could facilitate the synthesis of putrescine from arginine. Interestingly, we also noted a spermidine synthase gene expressed by *Dialister* and *Megasphaera* in BV, which can convert putrescine to another polyamine, spermidine. Although *Dialister* is a minor constituent of our samples, it has been detected in vaginal studies and associated with diverse microbiota profiles indicative of BV [1,7]. This could indicate that production of odor is a cooperative process depending on the particular microbiota composition. Indeed, odor is not universally detected in women who are BV-positive by Nugent score [40], and previous studies suggest that particular taxa are more associated with odor [3,41]. Symbiotic relationships may be common in the vaginal environment, as another has been described where *G. vaginalis* produces amino acids that promote the growth of *Prevotella bivia*[42].

## Conclusions

**Figure 3:**
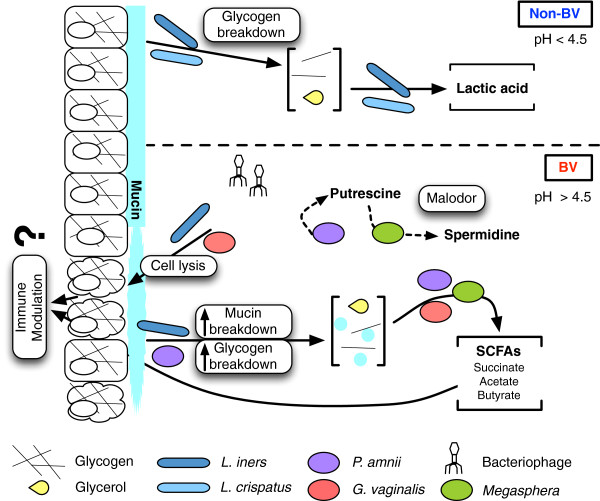
**Overview of predicted differential functions of the vaginal microbiota based on RNA-seq analysis. **The predominant lactobacilli present in the healthy condition results in a relatively simple environment where carbohydrates are converted to lactic acid. This results in a low pH and inhibition of the growth of other anaerobic organisms. The more complex microbial composition in bacterial vaginosis (BV) results in an increase in cell lysis, and an increase in carbohydrate availability. In this case, the carbohydrates are converted to succinate and other short-chain fatty acids (SCFAs) that can modulate host immunity, increase the pH and increase the reducing environment. Concomitant with this is an increase in bacteriophage load, and the potential for several organisms to cooperate to produce the malodorous compounds found in some cases of BV.

We used a novel method to characterize the conserved differences between the meta-transcriptome of two healthy and two BV microbial communities. Despite having four distinct microbial populations, our results show that each condition displays a set of conserved metabolic capabilities that, while influenced by the organisms present, is largely conserved between samples of differing structure. A summary of major findings is presented in Figure 3. The predicted metabolic end-products are known to produce the acidic or strongly reduced environments associated with health or BV respectively. Surprisingly, glycerol may have an underappreciated role in this environment with expression profiles suggesting upregulation of pathways to use glycerol as a carbon source under BV conditions. The source of glycerol is unknown, but it is an abundant component of mammalian and bacterial cell membranes, and extracellular glycerol levels are used in clinical practice to measure loss of cellular integrity. Thus, the rupture of cells by cytolysins expressed by *L. iners* and *G. vaginalis*, as well as lipase activity could release glycerol and glycerol components into the environment.

The BV-associated organism *P. amnii* showed enhanced glycogen extraction during BV, as well as the ability to synthesize its own glycogen. We posit a possible symbiotic relationship between the BV-associated organisms in our samples for the production of polyamines responsible for vaginal odor during BV. We additionally found that the vaginal microbiota respond to bacteriophage populations differently in healthy and BV conditions. Several longitudinal studies show rapid unexplained shifts in the vaginal microbiota [4,9], and one study suggested that phage have a role in the depletion of lactobacilli during episodes of BV [43]. This idea is supported by a recent study of viruses in the human fecal microbiota, which proposed that phage populations may be markers of change in the bacterial community [44].

We describe a different expression profile of *L. iners* in health and BV. The persistence of *L. iners* under these drastically differing environments (different pH, bacterial population composition, and bacterial load) could be due to its ability to respond and regulate its genomic functions. We suggest some of these regulated functions, such as specific carbohydrate uptake, bacteriophage defense, and a cytolysin, could be adapted for survival during episodes of BV. Some expressed functions were attributed to a subset of the *L. iners* genomes. Strain-specific functions of the microbiota, often overlooked by current 16S rRNA sequencing studies, can be extracted using function-based sequencing. Further investigation into the role of *L. iners* as a passive or active participant in the etiology and pathology of BV could help us understand this highly prevalent condition.

Overall, examining the meta-vaginal transcriptome has highlighted the complexity of this environment. The large difference in bacterial function between the two conditions emphasizes our need to better understand the interactions between species in the context of the rest of the community and of the host. It is likely that examination of additional microbiota types associated with health and BV will identify similar broad patterns, but will provide additional insights into the association between host symptoms and the action and products of the microbiome.

## Methods

### Clinical samples

Premenopausal women between the ages of 18 to 40 years were recruited at the Victoria Family Medical Center in London, Canada. The Health Sciences Research Ethics Board at the University of Western Ontario granted ethical approval for the study. Participants were excluded from the study if they had reached menopause, had a urogenital infection other than BV in the past 6 months, were pregnant, had a history of gonorrhoea, chlamydia, estrogen-dependent neoplasia, abnormal renal function or pyelonephritis, were taking prednisone, immunosuppresives or antimicrobial medication, or had undiagnosed abnormal vaginal bleeding. Participants were asked to refrain from oral or vaginal intercourse and consuming probiotic supplements or foods for 48 hours prior to the clinical visit. No participants were menstruating at time of the clinical visit. After reviewing details of the study, participants gave their signed informed consent before the start of the study. Vaginal swabs were collected from four women: two with BV and two considered to have a non-BV vaginal microbiota (N) as diagnosed by Nugent scoring [45], and vaginal *p*H (Additional file 1: Table S1). A nurse obtained vaginal samples for RNA-seq using a Dacron polyester-tipped swab rolled against the mid-vaginal wall and immediately suspended in RNAprotect (Qiagen, Valencia, CA, USA) containing 100 ug/ml rifampicin. A second swab collected in the same way was smeared onto a slide and air-dried for Nugent scoring [45] of bacterial vaginosis (Nugent scoring is presented in Additional file 1: Table S1). Vaginal pH was measured using the pHem-alert applicator (Gynex, Redmond, WA, USA). Samples for RNA extraction were incubated at room temperature for at least 10 minutes (to a maximum of 3 hours), and then centrifuged before discarding the supernatant and freezing the remaining pellet at 80°C. Lysis and RNA extraction were performed within 3 weeks of storage.

### RNA isolation, mRNA enrichment, and sequencing

Cell pellets were lysed in a 700 ul solution of 20 mg/mL lysozyme and 50 U/mL mutanolysin for 20 minutes at 37°C with periodic vortexing. After lysis, the samples were centrifuged (5500 · g for 15 minutes) and the supernatant was discarded. The remaining pellet was used for RNA extraction by TRIzol (Invitrogen, Grand Island, NY, USA) according to the manufacturer’s protocol. After RNA isolation by TRIzol, 8 to 9 ug of total RNA was used for rRNA depletion using a single round of MICROB*Express* (Ambion, Grand Island, NY, USA) according to the manufacturer’s protocol. RNA quality and subsequent rRNA depletion was verified before and after the MICROB*Express* treatment by the Agilent 2100 Bioanalyzer (Bioanalyzer results before and after MICROB*Express* treatment shown in Additional file 1: Figure S1). Samples were DNase-treated with the TURBO DNA-free kit (Ambion, Grand Island, NY, USA). Samples were sent to the Toronto Center for Applied Genomics (TCAG, Toronto, Ontario, Canada) for library preparation and sequencing by ABI SOLiD 4.

### Reference sequence library and mapping

A total of 110 accessions representing 103 organisms (of 31 genera, and 63 species) isolated from or detected in the vagina were included in a reference sequence set for mapping (Additional file 1: Table S4). The dataset represents the partial or complete genomes available from the NCBI database as of March 2011. These 234,991 sequences (including 230,031 coding sequences) were clustered by sequence identity (95% nucleotide identity over 90% sequence length) using CD-HIT-EST [46] to remove redundancy in the reference mapping set. A representative sequence (refseq) from each of the resulting 163,014 clusters was used to build a Bowtie [47] colorspace reference library for mapping the RNA-seq reads. Reads mapped uniquely by Bowtie to a coding refseq were included in the differential expression analysis (all other unmapped reads were discarded). Reads were trimmed from the 3^′^ end to 40 nt, and up to three mismatches were allowed. Reads with equal best hits were mapped at random to one of the locations.

### Functional assignment of refseqs

SOLiD colorspace format precludes direct functional assignment of sequenced reads, and therefore, annotations were assigned to the refseqs following mapping. Amino acid translations of predicted coding sequences were compared to the COG database [48] by rps-blast using an e-value cutoff of 1e-3 to assign a COG function. SEED Subsystems [49] were similarly assigned by blastp with an e-value cutoff of 1e-3. The KEGG Automatic Annotation Server (KAAS) [50] was used for annotation of enzyme functions (KOs) and mapping of metabolic pathways. All database comparisons and annotations were performed on the data available as of April 2011.

### Statistical analyses for differential expression

We used the ALDEx R package version 1.3.0 (Fernandes *et al*., unpublished work, http://code.google.com/p/aldex) for differential expression analysis. Refseqs belonging to *L. iners* were used to evaluate differential gene expression at the single organism level. For differential expression of the meta-bacterial community we modified the ALDEx package to group refseqs into higher functional levels (SEED subsystem4, and KEGG KO assignments). For genes or functional groups to be considered differentially expressed, we required a log_2_ relative difference (Δ*R*) of at least 2.0 for function-level analysis and at least 1.5 for *L. iners* gene-level analysis. To be considered differential we also allowed less than 1% of the distributions between the two conditions overlap. In the ALDEx output, this is defined as the quantile of 0 and has the symbol $\Delta A_{0}^{Q}$ ≤ 0.01. A brief description of the statistical framework is in the supplemental materials and is presented in Additional file 1: Figure S9.

### CRISPR spacer analysis

CRISPR spacer sequences were downloaded from the CRISPRs database (http://crispr.u-psud.fr/crispr/) and RNA-seq reads were mapped to these sequences as described for refseq mapping.

### *cpn60* reference mapping

Sequences belonging to the *cpn60* gene were downloaded from cpnDB [16] and were manually curated to remove redundant sequences (sequences with 100% identity to another sequence in the database), so that one representative sequence remained. RNA-seq reads were mapped to this reference set as described for refseq mapping.

## Abbreviations

ALDEx: analysis of variance-like differential expression (a software package implemented in R); AV: aerobic vaginitis; BV: bacterial vaginosis; CDC: cholesterol-dependent cytolysin; CDS: Coding DNA sequence; COG: clusters of orthologous groups; CRISPR: Clustered Regularly Interspaced Short Palindromic Repeats; GL: Glycerolipid; glpF: glycerol facilitator; Gly: Glycerol, Gly-3P, Glycerol-3-phosphate, GPL, Glycerophospholipid; KAAS: KEGG Automatic Annotation Server; KEGG: Kyoto Encyclopedia of Genes and Genomes; N: non-BV; PTS: phosphotransferase transport system; refseq: reference sequence; subsys: subsystem (referring to SEED level subsystems); Sus: starch utilization system; TCA: tricarboxylic acid.

## Competing interests

Gregor Reid holds US patent US7829079 ‘*Lactobacillus iners* for the enhancement of urogenital health’.

## Authors’ contributions

JMM prepared and processed samples for RNA extraction and rRNA depletion, performed data analysis including mapping RNA, performed differential analysis, functional annotation, prepared figures and tables, and wrote the manuscript. ADF developed ALDEx (statistical analysis for differential expression implemented as a software package in R), and wrote statistical methodology descriptions. JMDB aided functional analysis and provided supplemental Additional file 1: Figures S7 and S8. JAH coordinated patient recruitment and clinical data collection. GR performed study design and coordination, and wrote the manuscript. GBG conceived and designed experiments, performed data analysis and interpretation, and wrote the manuscript. All authors read and approved the final manuscript.

## Authors’ information

Gregor Reid and Gregory B Gloor, joint senior authors.

## Supplementary Material

Additional file 1 **Supporting information (PDF). **Contains Supporting supporting text describing the statistical methodology, the supporting figure and table headers and legends, Supporting Figures S1 to S10, and Supporting supporting Tables S1 to S6. Click here for file

Additional file 2: Table S8 Summary for Lactobacillus iners refseqs. Click here for file

Additional file 3: Table S9 Analysis of variance-like differential expression (ALDEx) ouptut for subsys4 functions. Click here for file

Additional file 4: Table S10 Analysis of variance-like differential expression (ALDEx) ALDEx output for KEGG KO functions. Click here for file

Additional file 5: Table S11 Lookup for Figure S7A functions (healthy, non-BV (N) condition). Click here for file

Additional file 6: Table S12 Lookup for Figure S7B functions (bacterial vaginosis (BV) condition). Click here for file

Additional file 7: Table S13 Lookup for Figure S8A functions (healthy, non-BV (N) condition). Click here for file

Additional file 8: Table S14 Lookup for Figure S8B functions (bacterial vaginosis (BV) condition). Click here for file

Additional file 9: Table S7 Reads mapped to refseqs and annotations. Click here for file
