# Supplementary material for: Comparative meta-RNA-seq of the vaginal microbiota and differential expression by Lactobacillus iners in health and dysbiosis
Source: Microbiome. 2013 Apr 12;1:12. doi: 10.1186/2049-2618-1-12 (PMC3971606; doi:10.1186/2049-2618-1-12)
Supplement: Additional file 1 — Supporting information (PDF). Contains Supporting supporting text describing the statistical methodology, the supporting figure and table headers and legends, Supporting Figures S1 to S10, and Supporting supporting Tables S1 to S6. [file 2049-2618-1-12-S1.pdf]

Statistical methodology for ANOVA-Like Differential Expression (ALDEx) analysis

The number of reads obtained for given gene in an RNA-seq dataset convey information about the relative contribution of that gene to the total number of reads, but conveys no information about the absolute expression level of the gene. Furthermore, RNA-seq datasets contain many genes with zero read counts (e.g. [1-4]) due to sparse sampling. The method described below explicitly accounts for the the proportional nature of the data and the probability that genes with 0 read counts actually represent non-expressed genes as opposed to those sampled with insufficient sequencing depth. The overall approach was to estimate the technical variance for each gene in each sample, and to identify as differential those genes where the separation between conditions was substantially larger than could be explained by technical variance.

We assumed that the read count, n, for each gene was sampled from a multinomial Poisson process of rate λ, i.e., $\{[n_1,n_2,\ldots]|n\}$~Multinomial$(p_1,p_2,\ldots|n)$ where the proportion for each gene can be represented by $p_i=\lambda_i/\sum_k \lambda_k$. The set of proportions p_i was estimated directly from the set of counts n_i using standard Bayesian techniques [5]:the posterior distribution of $[p_1,p_2,\ldots]$ was inferred as the product of the multinomial likelihood with a Dirichlet$(\frac{1}{2},\frac{1}{2},\ldots)$ prior. We generated 2048 instances from this distribution to estimate the technical variance inherent in tag-sequencing data [6]. Dirichlet-distributed random variates have the property that none of the inferred proportions are exactly 0, and are constrained to enforce conservation of proportion such that $\sum_k p_k=1$. The distribution also encapsulates the precision of estimating the proportion as a function of the total number of

read counts. All resulting operations were performed on the set of 2048 instances drawn from the Dirichlet samples rather than summary statistics because of the extreme non-normality that can arise when the read counts are small or 0.

Values from proportional distributions cannot be independent because these distributions are constrained to have a constant sum. Therefore, an increase in one or more proportions induces a concomitant decrease in one or more other proportions and *vice versa* [7]. Fortunately, the isometric log transformation [8] has been developed to transform the proportional values into linearly independent components. The procedure for a set of m proportions is simply to take component-wise logarithms and then to subtract the mean value of the resulting components. This results in the transformation of $p \mapsto q$, i.e., $q_i = \log(p_i) - 1m \sum_{k=1}^{m} \log(p_k)$. When base 2 logarithms are used, the adjusted log-proportion values q_i correspond exactly to “fold-based” abundances. This transformation has two major effects [8]. First, it removes the possibly-large multivariate statistical bias introduced by the log-transformation. Second, the covariance induced in the adjusted log-proportions q_i can be shown to be inversely proportional to the number of genes considered. For high-throughput data where thousands of genes are simultaneously considered, this induced covariance approaches zero. Although still not independent since $\sum_k q_k = 0$, carefully-constructed analyses can exploit this near-zero covariance in order to simplify numerical computations.

In the description of the method that follows, the individual genes using *i*, the different conditions using *j* and the different replicates of a condition using *k*. We use medians rather than means throughout to mitigate the effects of heavy tails and skewness that are prevalent in the distributions obtained from genes with low or 0 read-counts. Recall that we are dealing with Dirichlet-distributed random variates rather than point estimates for each gene in each sample in each condition. Similarly, the set of q_{ijk} are random variables as they are simple functions of the those Dirichlet-distributed variables. We now calculate the following random variable mixtures.

- The within-condition mixture distribution

$$W(i,j) = \sum_{k=1}^{K_j} q_{ijk}$$

- The absolute difference for gene *i* between-conditions *j* and *j'*

$$\Delta_A(i,j,j') = W(i,j) - W(i,j')$$

- The between sample, within-condition difference

$$\Delta_W(i,j) = \max_{k \neq k'} |q_{ijk} - q_{ijk'}|$$

- The relative effect-size between conditions

$$\Delta_R(i,j,j') = \Delta_A(i,j,j') \,/\, \max\{\Delta_W(i,j), \Delta_W(i,j')\}$$

The distribution of $W(i,j)$ is termed the "within-condition distribution" of gene i within condition j. Note that the "max" operator in the definition of Δ_W makes it a conservative representative of the pooled within-condition variance across conditions. The distribution of Δ_A is termed the "between-condition" distribution. Since the distributions of Δ_A, Δ_W, and Δ_R are estimated from multiple independent Monte Carlo realizations of their underlying Dirichlet-distributed proportions for all genes i simultaneously, they intrinsically obey the requisite $\sum_i q_{ijk}$=0 constraint. These distributions are thus unaffected by differing total-read counts.

The distributions of these random variables can be fairly broad or possess heavy tails especially when the corresponding read counts are low or 0. Thus, we summarize them by their quantiles with the notation: Δ_A^{50}, and Δ_A^{1-99} denoting the median, and 1–99% quantiles for Δ_A, and so on for the others. We further identify the quantile of zero in the distribution of Δ_A as $\Delta_A^{Q_0}$. We use a symmetric variant of $\Delta_A^{Q_0}$ such that $\Delta_A^{Q_0}$≤0.5. note that throughout we used the full distribution of Dirichlet-distributed values. A graphical depiction of our approximate-ANOVA is depicted in Figure S9.

Differential expression was determined using the thresholds for Δ_R≥2 and $\Delta_A^{Q_0}$≤0.01. A value of Δ_R≥2.0 means that the difference between conditions is at least twice widest within-condition variance, while $\Delta_A^{Q_0}$≤0.01 implies fewer than 1% of the values in the $W(i,j)$ and $W(i,j')$ distributions overlap. Changing threshold values corresponds to adjusting the

stringency of a hypothesis test, with concomitant trade-off in resultant Type-I and Type-II errors.

ALDEx is avaialble from: http://code.google.com/p/aldex

References

1. Marioni JC, Mason CE, Mane SM, Stephens M, Gilad Y: **RNA-seq: An assessment of technical reproducibility and comparison with gene expression arrays.** *Genome Research* 2008, **18**(9)**:**1509-1517.
2. Polymenakou PN, Lampadariou N, Mandalakis M, Tselepides A: **Phylogenetic diversity of sediment bacteria from the southern Cretan margin, Eastern Mediterranean Sea.** *Syst Appl Microbiol* 2009, **32**(1)**:**17-26.
3. Crawford JE, Guelbeogo WM, Sanou A, Traore A, Vernick KD, Sagnon N, Lazzaro BP: **De novo transcriptome sequencing in Anopheles funestus using Illumina RNA-seq technology.** *PLoS One* 2010, **5**(12)**:**e14202.
4. Rosenthal AZ, Matson EG, Eldar A, Leadbetter JR: **RNA-seq reveals cooperative metabolic interactions between two termite-gut spirochete species in co-culture.** *ISME J* 2011, **5**(7)**:**1133-1142.
5. Jaynes ET, Bretthorst GL: *Probability Theory: the Logic of Science:* Cambridge, UK: Cambridge University Press; 2003.

6. La Rosa PS, Brooks JP, Deych E, Boone EL, Edwards DJ, Wang Q, Sodergren E, Weinstock G, Shannon WD: **Hypothesis testing and power calculations for taxonomic-based human microbiome data.** *PLoS One* 2012, **7**(12)**:**e52078.

7. Pearson K: **Mathematical Contributions to the Theory of Evolution.--On a Form of Spurious Correlation Which May Arise When Indices Are Used in the Measurement of Organs.** *Proceedings of the Royal Society of London* 1896, **60:**489-498.

8. Egozcue JJ, Pawlowsky-Glahn V, Mateu-Figueras G, Barceló-Vidal C: **Isometric Logratio Transformations for Compositional Data Analysis.** *Math Geol* 2003, **35**(3)**:**279-300.

Supporting Figure Legends and Table Header Descriptions

Figure S1

Agilent 2100 Bioanalyzer electropherograms showing bacterial RNA samples before and after rRNA depletion by MICROB*Express*. The large 16S and 23S peaks that make up much of the total RNA sample have been reduced by >99% after a single round of MICROB*Express*. The bioanalyzer output for sample N30 after treatment was no available, but rRNA depletion was validated before sequencing. Note the difference in the relative fluorescence scale (y-axis) between plots.

Figure S2

Heatmap representation of the mean log_2 expression of reference sequences (A) and the mean log_2 expression of the reference sequences grouped by SEED subsystem 4 (B) for each sample. The mean log_2 expression values were computed into a distance matrix using the 'manhattan' method in R. The values of the distance matrix are represented in the heatmaps, and pairs of samples closer to 0 are more similar in log_2 expression. Grouping reference sequences by function (B) results in a tighter clustering of samples within a condition (shorter branch lengths and lower distance values).

Figure S3

Select SEED subsystem strip plots showing enriched functions for *L. iners* differential expression analysis. Each point is a refseq belonging to *L. iners* and points are colored red (BV) or blue (N) if significantly differential in their respective conditions. Points are plotted

by the median log_2 difference in expression between conditions: the value is positive if the refseq is more highly expressed in BV, and negative if it is more highly expressed in N.

Figure S4

COG (Clusters of Orthologous Groups of proteins) function distribution for *L. iners* refseqs per sample. The first three plots show the fraction of reads belonging to each COG category for the *L. iners* pan-transcriptome ("all mapped reads"), the top 10% expressed refseqs, and the total differential reads. The final plot shows the "binned" number of differential genes in each COG category. The color legend below also applies to Figure 2 in the main manuscript

Figure S5

Strip plot summary of absolute expression differences for subsys4 functions binned by SEED functional categories. Differential expression analysis was performed using ALDEx for refseqs grouped by SEED subsystem 4. Each point represents one subsys4 functional group, and points are plotted by SEED subsystem level 1 (A), subsystem level 2 (B), or subsystem level 3 (C) categories. Points are colored blue (non-BV) or red (BV) if significantly differentially expressed between conditions.

Figure S6

Heatmap summary of the transcriptional effort (expression) by the bacterial community per SEED functional category. The expression of each subsys4 function as calculated by ALDEx (relative to the average of all subsys4 functions) was summed and divided by the

number of subsys4 per SEED subsystem level 1 (A), subsystem level 2 (B), or subsystem level 3 (C) categories.

Figures S7 and S8

Function/fraction plots showing the taxonomic assignment of the reads belonging to significantly differential functions. The paired barplots represent the two samples for one function. Shown are significantly differential functions of subsys level 3 for N condition (Figure S7A) and BV (Figure S7B). Significantly differential functions for KEGG analysis at pathway level (hier3) are shown for N condition (Figure S8A) and BV (Figure S8B). The colored legend for taxon assignments is presented in Figure 1 of the main manuscript text. A table lookup for each set of plots lists the functions plotted for each category (Tables S10 to S13).

Figure S9

Outline of the statistical analysis of meta-transcriptome data (AF et al, PLoS). Step 1, reads are binned according to SEED, KEGG or COG function. In the example, there are two samples from two conditions B (red) and N (blue). Step 2, read values are converted to proportions by drawing from the Dirichlet distribution, 2048 instances are collected to estimate technical variance due to sampling and sequencing. These proportions are log-transformed and the mean expression in each sample is subtracted from each value. This transformation converts the values into linearly independent values and the mean expression value of each sample is now 0. The difference between the within-condition distributions is determined by subtraction, giving a distribution of differences represented

below. The adjacent number represents the median difference. In step 3, distributions for the samples in each condition are merged into a joint distribution and the difference between conditions is determined by subtraction. Finally, as shown in step 4, the between-condition difference is divided by the larger of the within-condition differences. This results in a distribution that measures the difference between conditions scaled by the difference between samples in a condition. The method identifies genes that exhibit consistency of expression between samples within conditions and large differences between conditions. (See supporting text for a full description).

Figure S10

This figure is a complement to Figure 2: Circular representation of RNA-seq data for the *Lactobacillus iners* pan-transcriptome. RNA-seq reads were mapped to 12 available *L. iners'* genomes (listed in Table S3) after clustering redundant coding sequences by nucleotide identity (see Materials and Methods). Breaks in the circle separate blocks of contiguous coding sequences (CDS) ordered by scaffolding on the genome assemblies (Ring ①). The height of the plot represents how many of the 12 genomes the CDS occurs. The first contiguous block (dark blue) are the 954 CDS present in all genomes and are considered the "core" *L. iners* gene set. Ring ② shows the COG color-coded function of each CDS. Ring ③ The differential expression of each CDS between Non-BV and BV samples. The absolute height of the bar shows the fold-change ($\log_2$) in expression (positive for up BV, and negative for up non-BV). Bars are colored if significantly differential (red for BV and blue for non-BV). Regions of interest are labeled in the centre of the circle. Rings ④ ,⑤, and ⑥ show a heatmap representation of the median CDS expression in BV, Non-BV, and *L. iners*

grown to mid-log phase in MRS broth respectively. Darker blue represents higher expression. Regions of interest noted in the results are marked and lettered as follows: A) Glycogen metabolism and uptake, B) Inolysin (CDC), C) PTS mannose-type, D) Glycerol metabolism, E) CRISPRs.

Tables S7 to S10 headers descriptions

refseq_id the identifier sequence for clustered CDS; **length** the refseq length in nt; **annotation** the refseq annotation from NCBI; **common_taxonomy** the lowest taxonomic level common to all sequences in the refseq cluster (represented by a single refseq ID); **cluster** the CD-HIT cluster number for the refseq; **no_seqs** the number of sequences in the CD-HIT cluster; **N4_total** the total number of reads mapped to this refseq (each sample in its own column); **subsys4 or kegg** the assigned SEED subsystem level 4 or kegg (KO number) function for the refseq; **expression.** the ALDEx-calculated median expression value for the refseq or function. This is relative to the average expression of all refseqs with the average expression being 0. A negative value represents expression below average, while a positive value is expression above average; **expression.within.N** or **B** The average median expression of the refseq within a condition (e.g. for N the average of **N4** and **N30** median expression); **expression.among.q50** the median expression across all samples (all conditions); **expression.sample.** median expression for the refseq/function in the indicated sample (N4, N30, B27, or B31); **difference.between.q50** median magnitude of change between conditions (median log2 fold change); **difference.within.q50** median maximum magnitude of change within conditions; **effect.q50** median relative difference; **criteria.significant** TRUE if **criteria.significance** is >= 0.01 (this is proportion of the

sample distributions that overlap between conditions); **criteria.meaningful** is TRUE if **criteria.meaning** is >= the cutoff chosen (this is the relative difference. A value of 2 ensures that the between-condition is at least a 4-fold larger than the within-condition difference); **sig.both** TRUE if criteria.meaningful and criteria.significant are both TRUE. These represent our significantly differntial genes/functions; **expression.D.q50** expression for a single sample of *L. iners* AB-1 grown in MRS broth

Figure S1

Total RNA	Enriched mRNA

N4

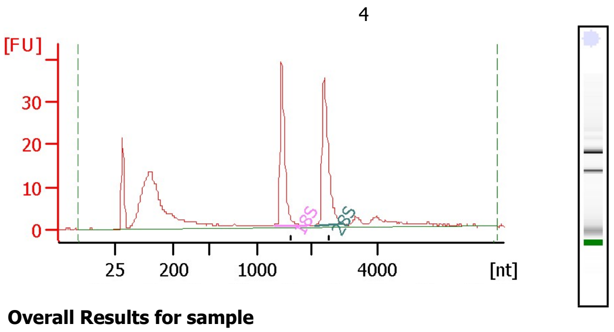

Overall Results for sample

RNA Area: 318.5
RNA Concentration: 99 ng/µl
rRNA Ratio [23s / 16s]: 1.2
RNA Integrity Number (RIN): 9.3 (B.02.07, Anomaly Threshold(s) manually adapted)

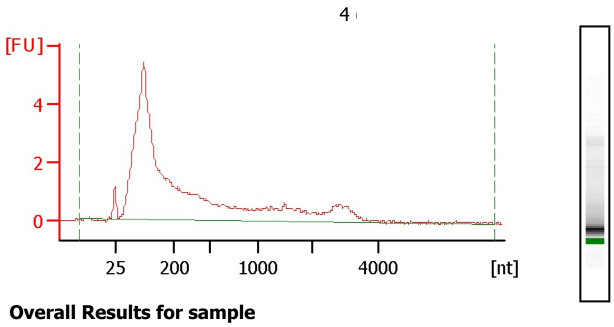

Overall Results for sample

RNA Area: 82.5
RNA Concentration: 213 ng/µl
rRNA Ratio [23s / 16s]: 0.0
RNA Integrity Number (RIN): 2.3 (B.02.07, Anomaly Threshold(s) manually adapted)

B27

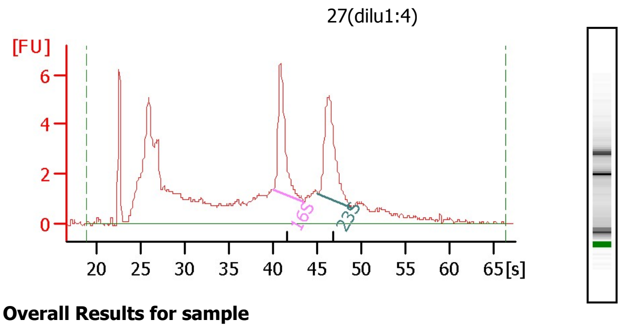

Overall Results for sample

RNA Area: 127.3
RNA Concentration: 106 ng/µl
rRNA Ratio [23s / 16s]: 1.1
RNA Integrity Number (RIN): 7.2 (B.02.07)

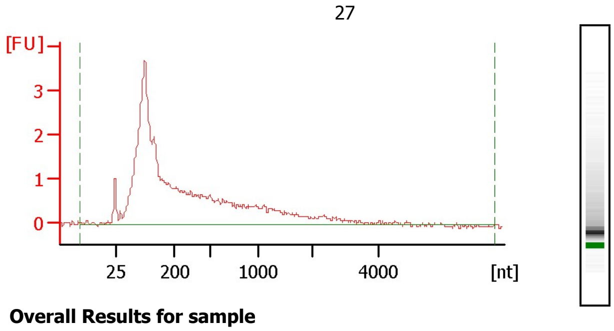

Overall Results for sample

RNA Area: 54.6
RNA Concentration: 141 ng/µl
rRNA Ratio [23s / 16s]: 0.0
RNA Integrity Number (RIN): 2.2 (B.02.07, Anomaly Threshold(s) manually adapted)

N30

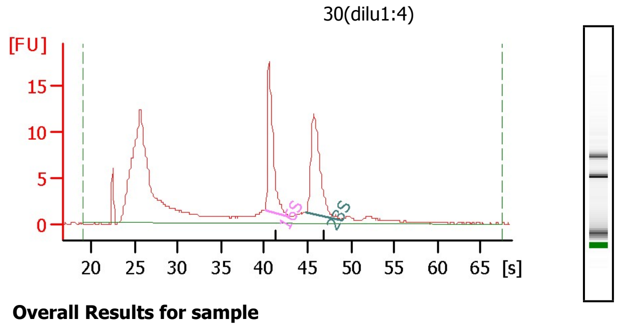

Overall Results for sample

RNA Area: 223.7
RNA Concentration: 186 ng/µl
rRNA Ratio [23s / 16s]: 1.0
RNA Integrity Number (RIN): 8 (B.02.07, Anomaly Threshold(s) manually adapted)

B31

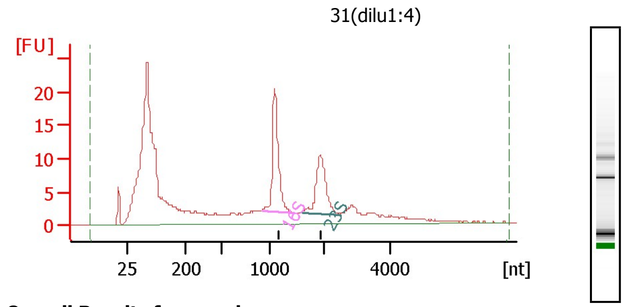

Overall Results for sample

RNA Area: 362.6
RNA Concentration: 301 ng/µl
rRNA Ratio [23s / 16s]: 0.7
RNA Integrity Number (RIN): 7.1 (B.02.07, Anomaly Threshold(s) manually adapted)

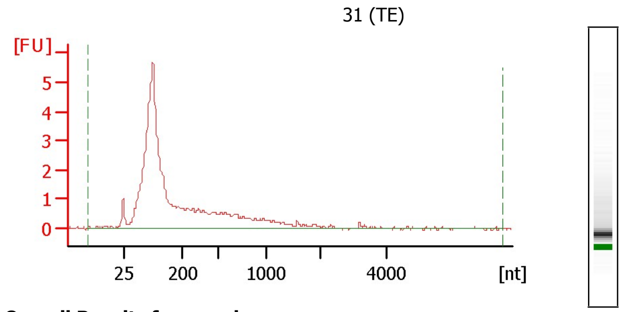

Overall Results for sample

RNA Area: 61.6
RNA Concentration: 159 ng/µl
rRNA Ratio [23s / 16s]: 0.0
RNA Integrity Number (RIN): 2.3 (B.02.07, Anomaly Threshold(s) manually adapted)

Figure S2

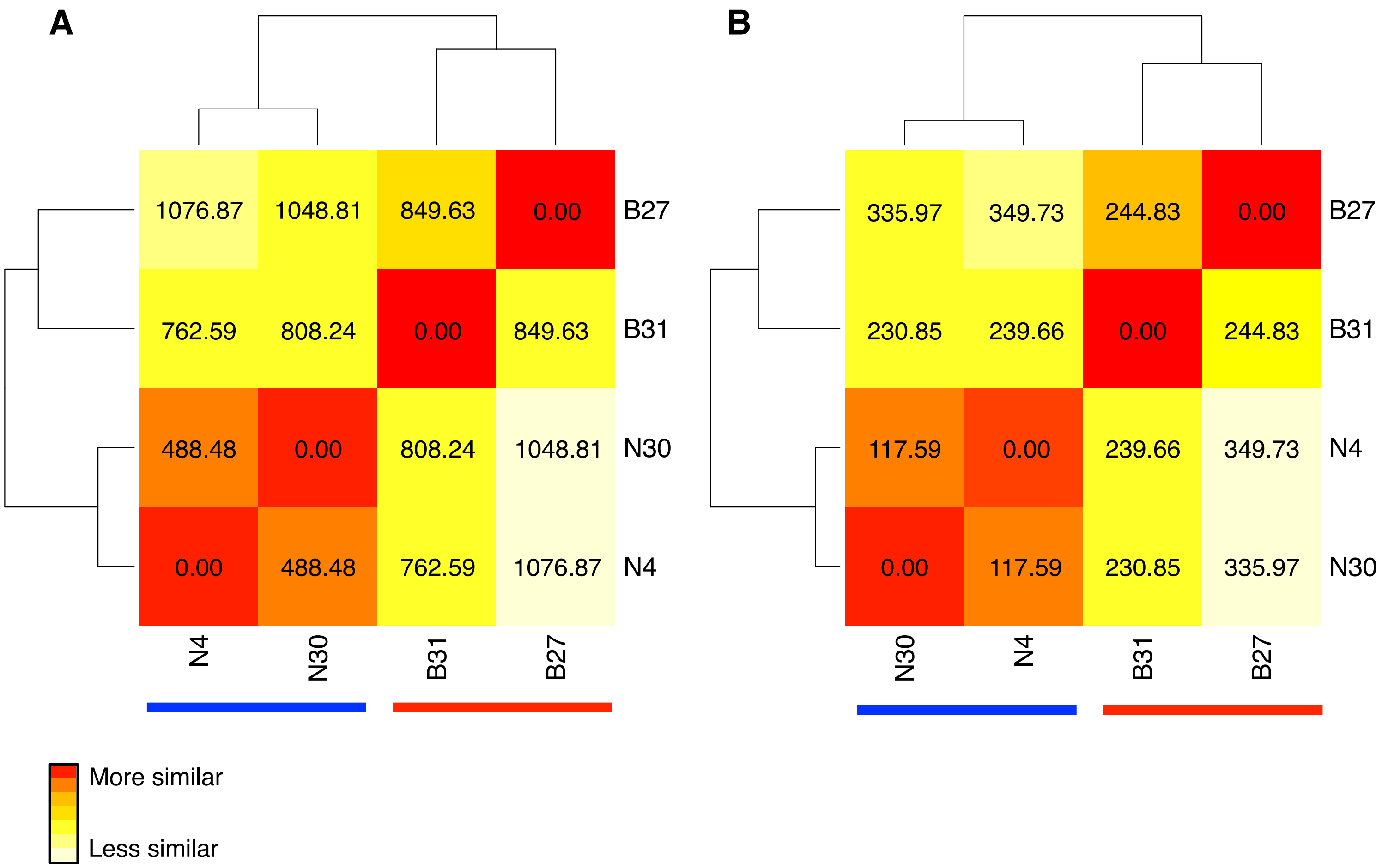
A
1076.87
1048.81
849.63
0.00
B27
762.59
808.24
0.00
849.63
B31
488.48
0.00
808.24
1048.81
N30
0.00
488.48
762.59
1076.87
N4
N4
N30
B31
B27
B
335.97
349.73
244.83
0.00
B27
230.85
239.66
0.00
244.83
B31
117.59
0.00
239.66
349.73
N4
0.00
117.59
230.85
335.97
N30
N30
N4
B31
B27
More similar
Less similar

Figure S3A

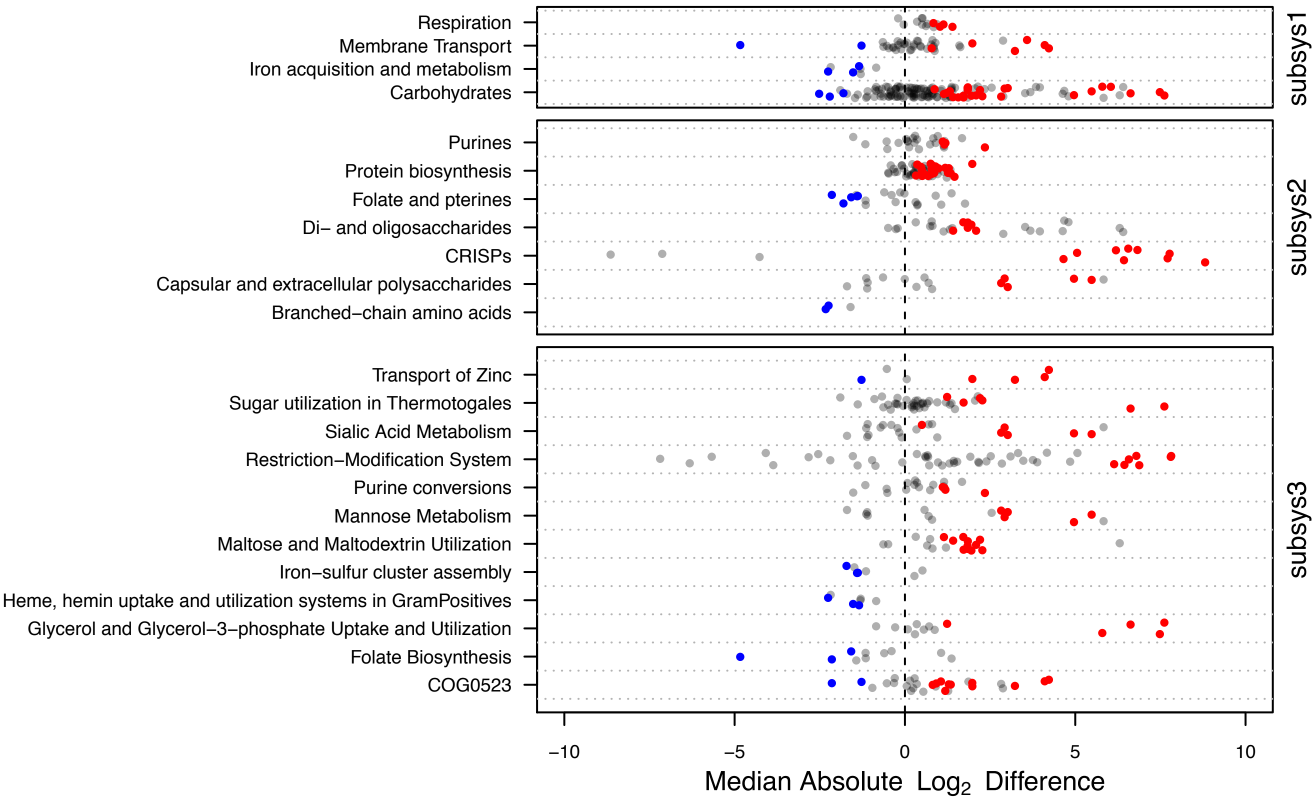
Respiration
Membrane Transport
Iron acquisition and metabolism
Carbohydrates
subsys1
Purines
Protein biosynthesis
Folate and pterines
Di– and oligosaccharides
CRISPs
Capsular and extracellular polysaccharides
Branched–chain amino acids
subsys2
Transport of Zinc
Sugar utilization in Thermotogales
Sialic Acid Metabolism
Restriction–Modification System
Purine conversions
Mannose Metabolism
Maltose and Maltodextrin Utilization
Iron–sulfur cluster assembly
Heme, hemin uptake and utilization systems in GramPositives
Glycerol and Glycerol–3–phosphate Uptake and Utilization
Folate Biosynthesis
COG0523
subsys3
–10
–5
0
5
10
Median Absolute Log2 Difference

Figure S4

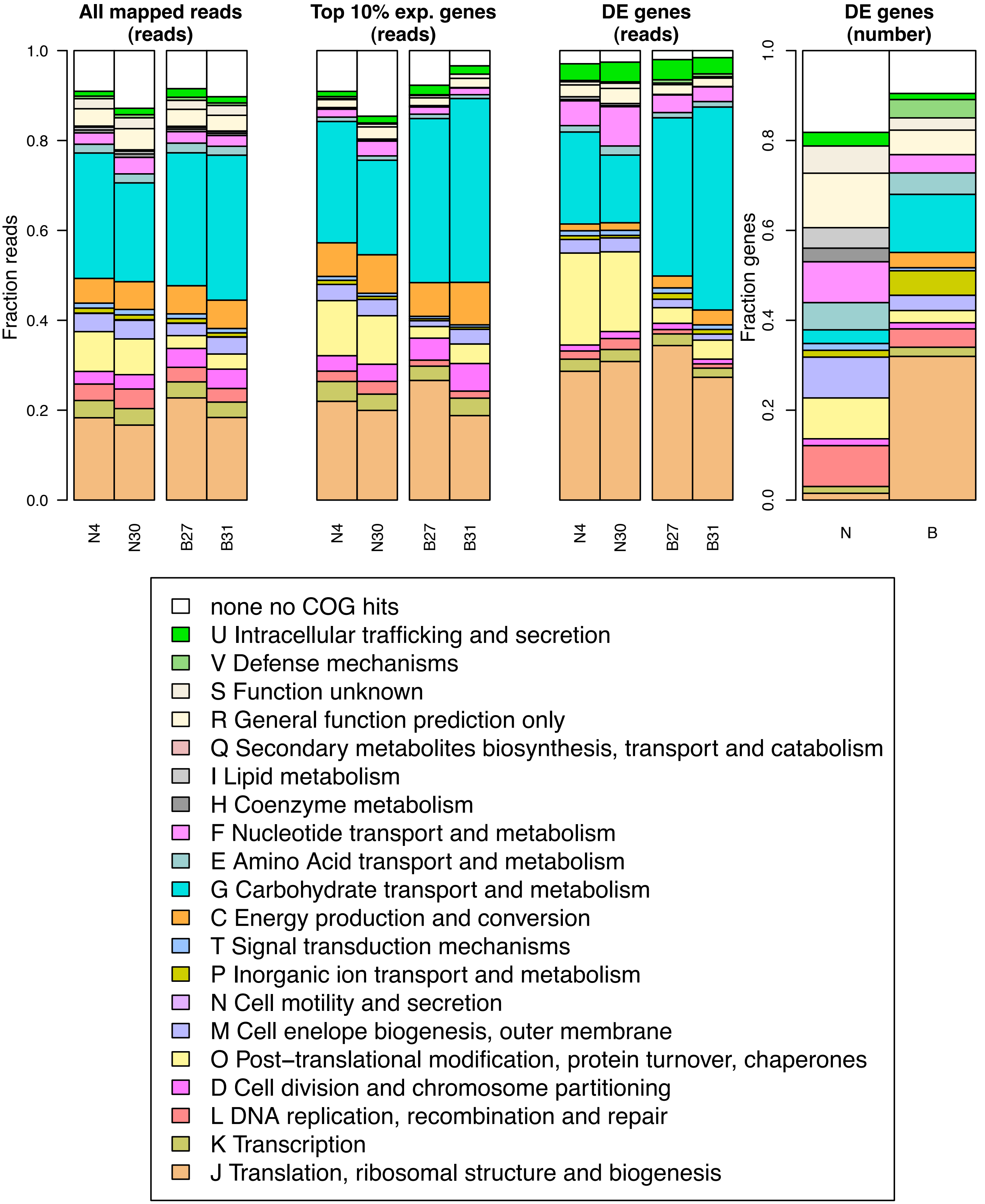

All mapped reads (reads)
Top 10% exp. genes (reads)
DE genes (reads)
DE genes (number)
Fraction reads
Fraction genes
0.0
0.2
0.4
0.6
0.8
1.0
N4
N30
B27
B31
N
B
none no COG hits
U Intracellular trafficking and secretion
V Defense mechanisms
S Function unknown
R General function prediction only
Q Secondary metabolites biosynthesis, transport and catabolism
I Lipid metabolism
H Coenzyme metabolism
F Nucleotide transport and metabolism
E Amino Acid transport and metabolism
G Carbohydrate transport and metabolism
C Energy production and conversion
T Signal transduction mechanisms
P Inorganic ion transport and metabolism
N Cell motility and secretion
M Cell enelope biogenesis, outer membrane
O Post−translational modification, protein turnover, chaperones
D Cell division and chromosome partitioning
L DNA replication, recombination and repair
K Transcription
J Translation, ribosomal structure and biogenesis

Figure 5A

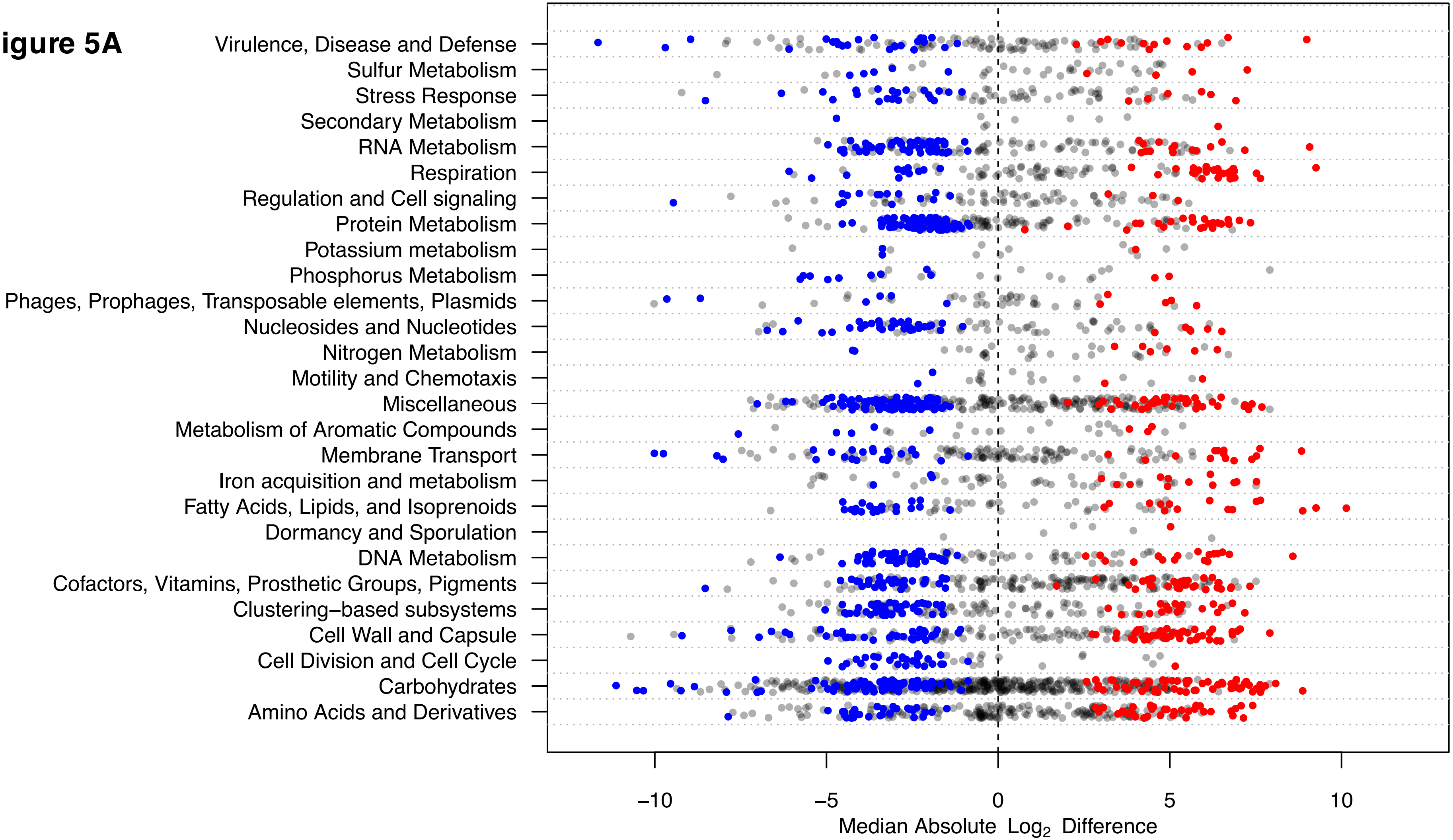
Virulence, Disease and Defense
Sulfur Metabolism
Stress Response
Secondary Metabolism
RNA Metabolism
Respiration
Regulation and Cell signaling
Protein Metabolism
Potassium metabolism
Phosphorus Metabolism
Phages, Prophages, Transposable elements, Plasmids
Nucleosides and Nucleotides
Nitrogen Metabolism
Motility and Chemotaxis
Miscellaneous
Metabolism of Aromatic Compounds
Membrane Transport
Iron acquisition and metabolism
Fatty Acids, Lipids, and Isoprenoids
Dormancy and Sporulation
DNA Metabolism
Cofactors, Vitamins, Prosthetic Groups, Pigments
Clustering–based subsystems
Cell Wall and Capsule
Cell Division and Cell Cycle
Carbohydrates
Amino Acids and Derivatives
−10
−5
0
5
10
Median Absolute Log_2 Difference

Figure 5B

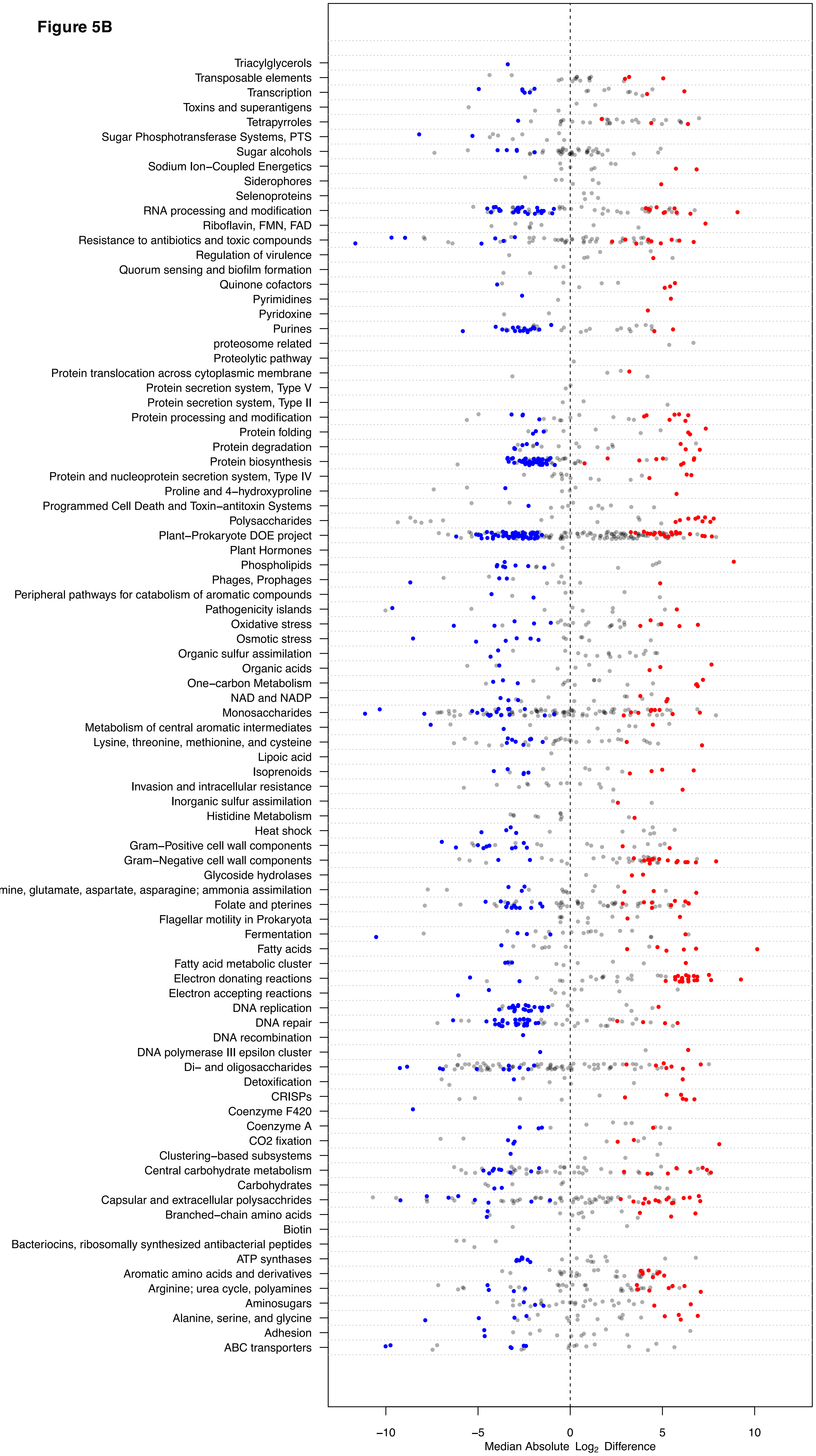

Triacylglycerols
Transposable elements
Transcription
Toxins and superantigens
Tetrapyrroles
Sugar Phosphotransferase Systems, PTS
Sugar alcohols
Sodium Ion−Coupled Energetics
Siderophores
Selenoproteins
RNA processing and modification
Riboflavin, FMN, FAD
Resistance to antibiotics and toxic compounds
Regulation of virulence
Quorum sensing and biofilm formation
Quinone cofactors
Pyrimidines
Pyridoxine
Purines
proteosome related
Proteolytic pathway
Protein translocation across cytoplasmic membrane
Protein secretion system, Type V
Protein secretion system, Type II
Protein processing and modification
Protein folding
Protein degradation
Protein biosynthesis
Protein and nucleoprotein secretion system, Type IV
Proline and 4−hydroxyproline
Programmed Cell Death and Toxin−antitoxin Systems
Polysaccharides
Plant−Prokaryote DOE project
Plant Hormones
Phospholipids
Phages, Prophages
Peripheral pathways for catabolism of aromatic compounds
Pathogenicity islands
Oxidative stress
Osmotic stress
Organic sulfur assimilation
Organic acids
One−carbon Metabolism
NAD and NADP
Monosaccharides
Metabolism of central aromatic intermediates
Lysine, threonine, methionine, and cysteine
Lipoic acid
Isoprenoids
Invasion and intracellular resistance
Inorganic sulfur assimilation
Histidine Metabolism
Heat shock
Gram−Positive cell wall components
Gram−Negative cell wall components
Glycoside hydrolases
mine, glutamate, aspartate, asparagine; ammonia assimilation
Folate and pterines
Flagellar motility in Prokaryota
Fermentation
Fatty acids
Fatty acid metabolic cluster
Electron donating reactions
Electron accepting reactions
DNA replication
DNA repair
DNA recombination
DNA polymerase III epsilon cluster
Di− and oligosaccharides
Detoxification
CRISPs
Coenzyme F420
Coenzyme A
CO2 fixation
Clustering−based subsystems
Central carbohydrate metabolism
Carbohydrates
Capsular and extracellular polysacchrides
Branched−chain amino acids
Biotin
Bacteriocins, ribosomally synthesized antibacterial peptides
ATP synthases
Aromatic amino acids and derivatives
Arginine; urea cycle, polyamines
Aminosugars
Alanine, serine, and glycine
Adhesion
ABC transporters
−10
−5
0
5
10
Median Absolute Log2 Difference

Figure 5C

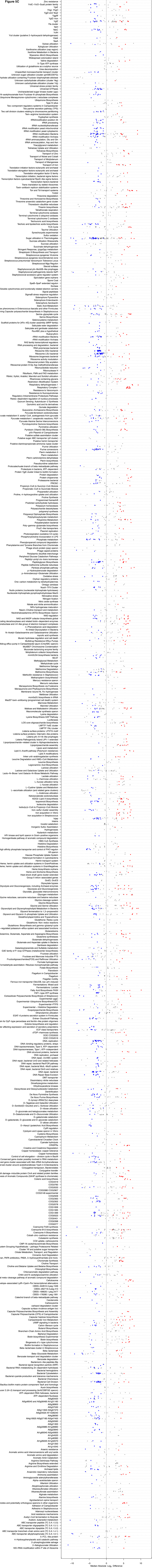

Figure S6A

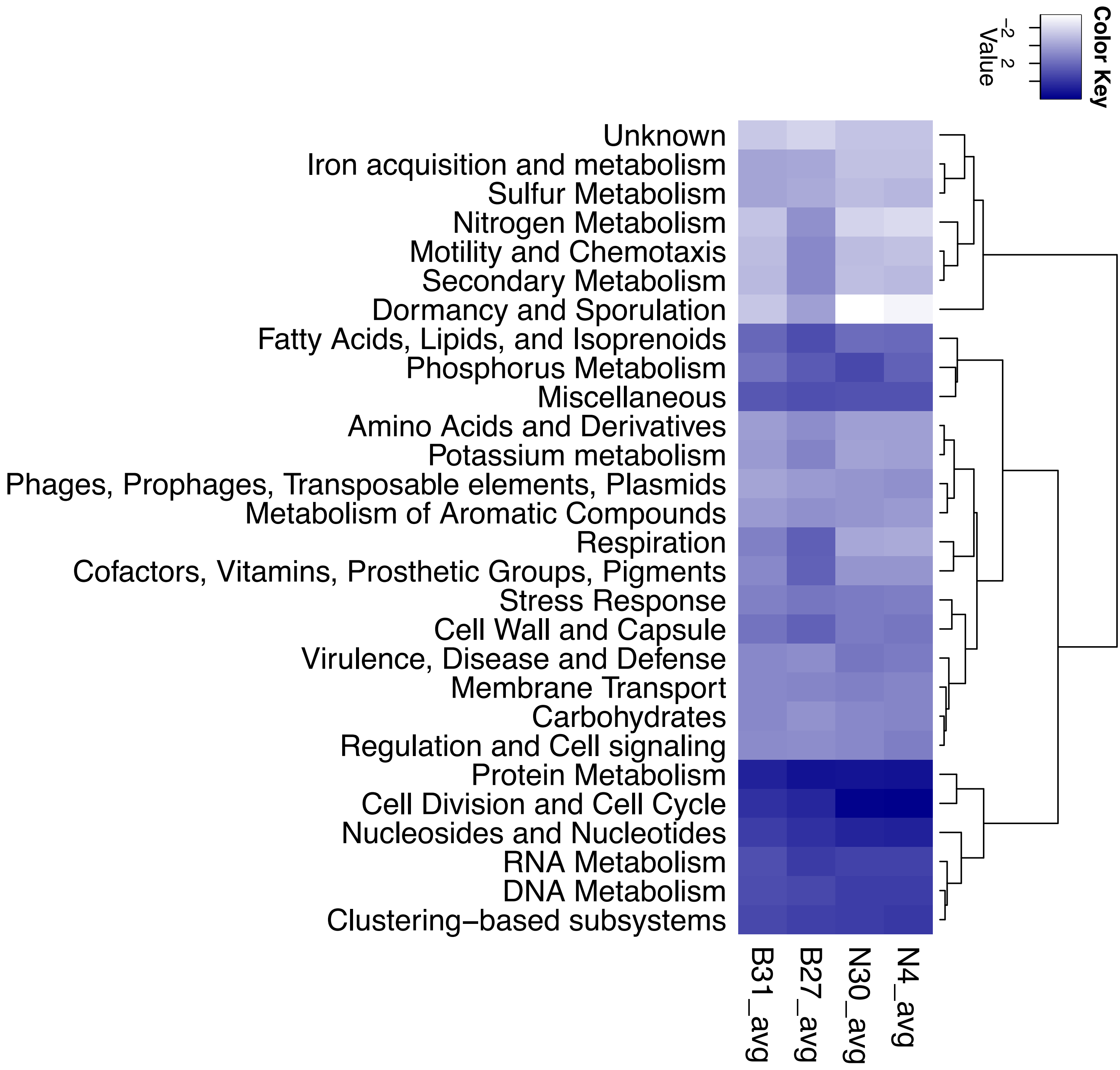

Color Key
Value
−2
2
Unknown
Iron acquisition and metabolism
Sulfur Metabolism
Nitrogen Metabolism
Motility and Chemotaxis
Secondary Metabolism
Dormancy and Sporulation
Fatty Acids, Lipids, and Isoprenoids
Phosphorus Metabolism
Miscellaneous
Amino Acids and Derivatives
Potassium metabolism
Phages, Prophages, Transposable elements, Plasmids
Metabolism of Aromatic Compounds
Respiration
Cofactors, Vitamins, Prosthetic Groups, Pigments
Stress Response
Cell Wall and Capsule
Virulence, Disease and Defense
Membrane Transport
Carbohydrates
Regulation and Cell signaling
Protein Metabolism
Cell Division and Cell Cycle
Nucleosides and Nucleotides
RNA Metabolism
DNA Metabolism
Clustering−based subsystems
B31_avg
B27_avg
N30_avg
N4_avg

Figure S6B

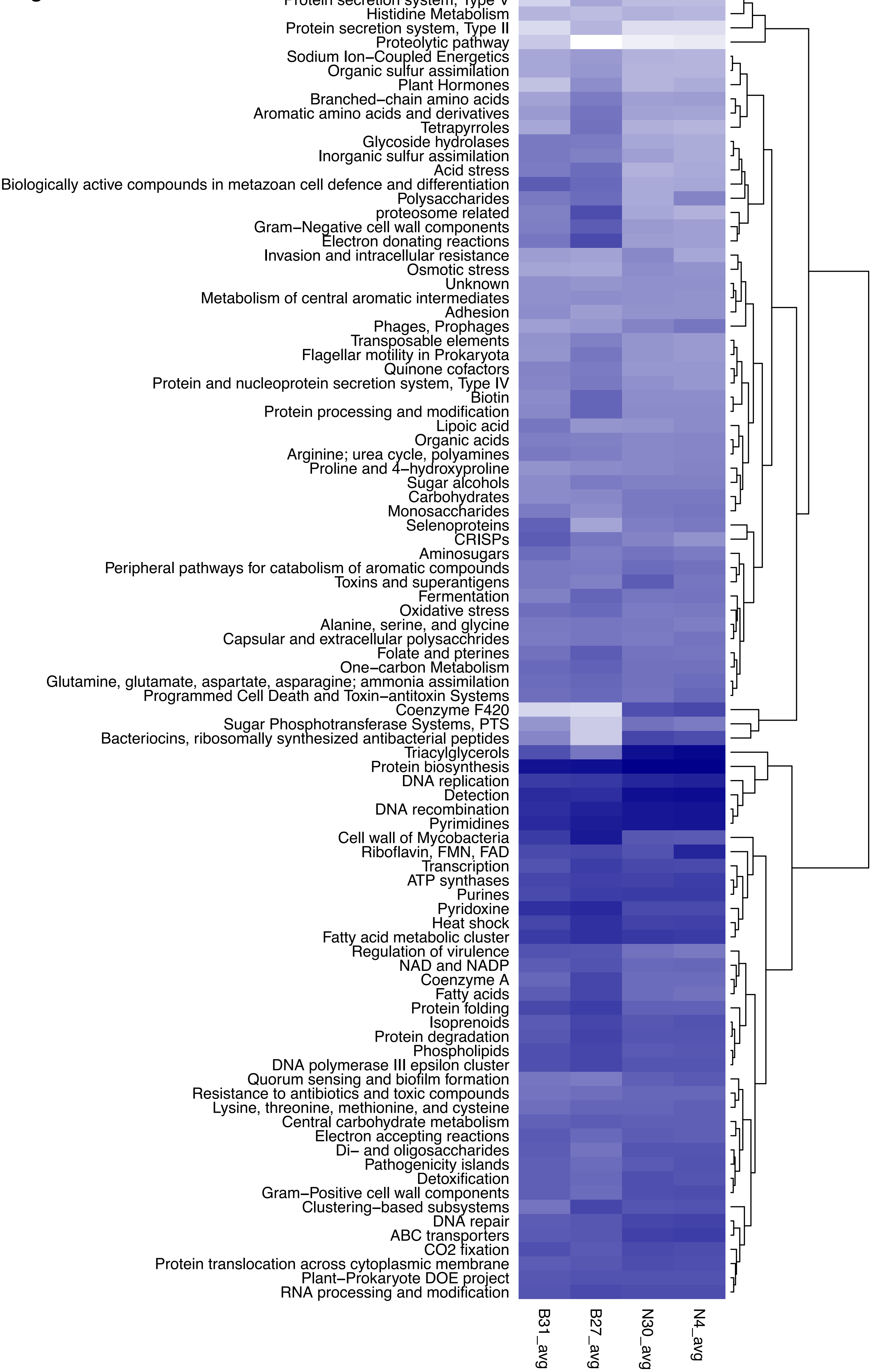
Siderophores
Protein secretion system, Type V
Histidine Metabolism
Protein secretion system, Type II
Proteolytic pathway
Sodium Ion−Coupled Energetics
Organic sulfur assimilation
Plant Hormones
Branched−chain amino acids
Aromatic amino acids and derivatives
Tetrapyrroles
Glycoside hydrolases
Inorganic sulfur assimilation
Acid stress
Biologically active compounds in metazoan cell defence and differentiation
Polysaccharides
proteosome related
Gram−Negative cell wall components
Electron donating reactions
Invasion and intracellular resistance
Osmotic stress
Unknown
Metabolism of central aromatic intermediates
Adhesion
Phages, Prophages
Transposable elements
Flagellar motility in Prokaryota
Quinone cofactors
Protein and nucleoprotein secretion system, Type IV
Biotin
Protein processing and modification
Lipoic acid
Organic acids
Arginine; urea cycle, polyamines
Proline and 4−hydroxyproline
Sugar alcohols
Carbohydrates
Monosaccharides
Selenoproteins
CRISPs
Aminosugars
Peripheral pathways for catabolism of aromatic compounds
Toxins and superantigens
Fermentation
Oxidative stress
Alanine, serine, and glycine
Capsular and extracellular polysacchrides
Folate and pterines
One−carbon Metabolism
Glutamine, glutamate, aspartate, asparagine; ammonia assimilation
Programmed Cell Death and Toxin−antitoxin Systems
Coenzyme F420
Sugar Phosphotransferase Systems, PTS
Bacteriocins, ribosomally synthesized antibacterial peptides
Triacylglycerols
Protein biosynthesis
DNA replication
Detection
DNA recombination
Pyrimidines
Cell wall of Mycobacteria
Riboflavin, FMN, FAD
Transcription
ATP synthases
Purines
Pyridoxine
Heat shock
Fatty acid metabolic cluster
Regulation of virulence
NAD and NADP
Coenzyme A
Fatty acids
Protein folding
Isoprenoids
Protein degradation
Phospholipids
DNA polymerase III epsilon cluster
Quorum sensing and biofilm formation
Resistance to antibiotics and toxic compounds
Lysine, threonine, methionine, and cysteine
Central carbohydrate metabolism
Electron accepting reactions
Di− and oligosaccharides
Pathogenicity islands
Detoxification
Gram−Positive cell wall components
Clustering−based subsystems
DNA repair
ABC transporters
CO2 fixation
Protein translocation across cytoplasmic membrane
Plant−Prokaryote DOE project
RNA processing and modification
B31_avg
B27_avg
N30_avg
N4_avg

Figure S6C

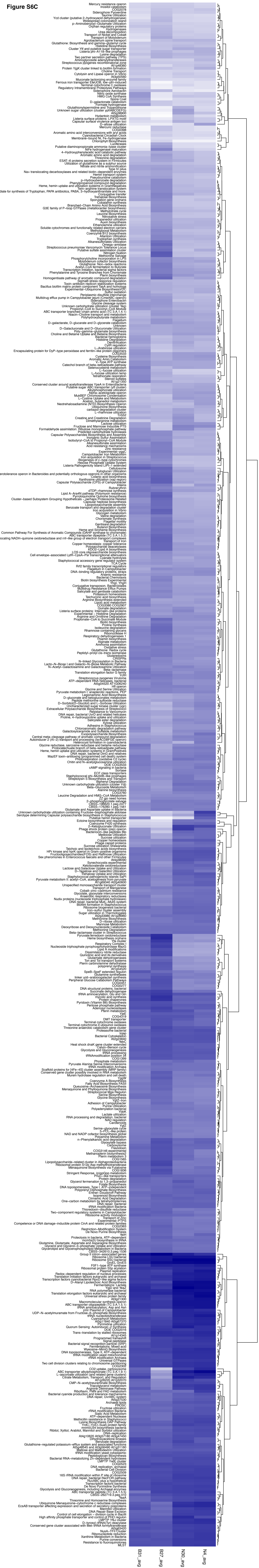

Figure S7A

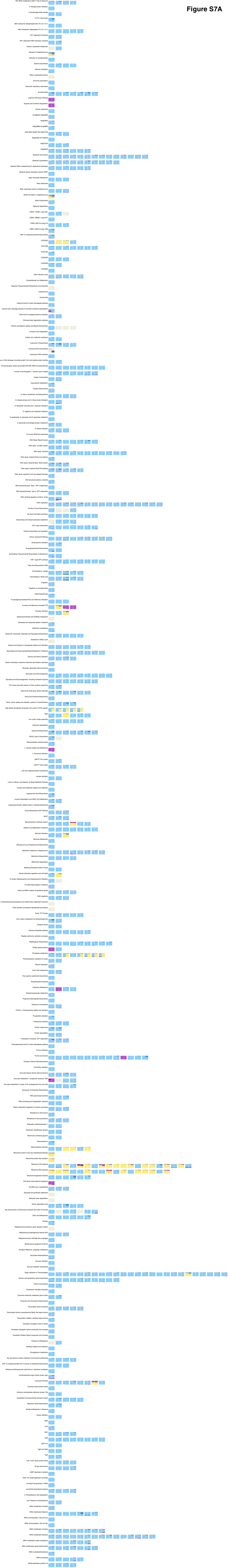

Figure S7B

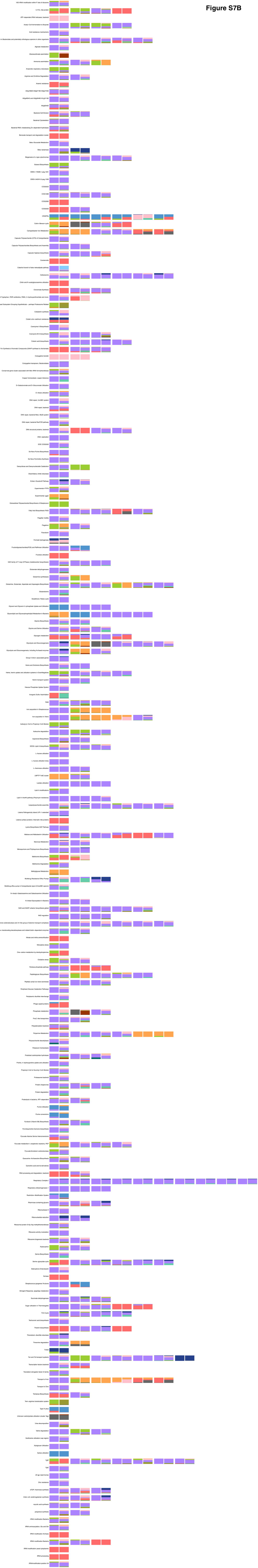

Figure S8A

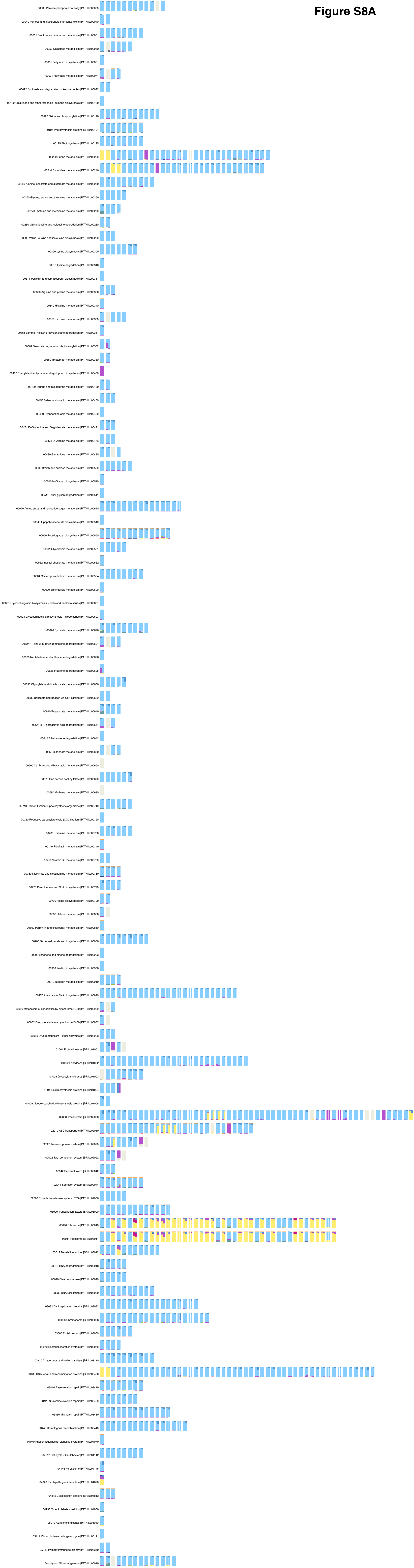
00030 Pentose phosphate pathway [PATH:ko00030]
00040 Pentose and glucuronate interconversions [PATH:ko00040]
00051 Fructose and mannose metabolism [PATH:ko00051]
00052 Galactose metabolism [PATH:ko00052]
00061 Fatty acid biosynthesis [PATH:ko00061]
00071 Fatty acid metabolism [PATH:ko00071]
00072 Synthesis and degradation of ketone bodies [PATH:ko00072]
00130 Ubiquinone and other terpenoid−quinone biosynthesis [PATH:ko00130]
00190 Oxidative phosphorylation [PATH:ko00190]
00194 Photosynthesis proteins [BR:ko00194]
00195 Photosynthesis [PATH:ko00195]
00230 Purine metabolism [PATH:ko00230]
00240 Pyrimidine metabolism [PATH:ko00240]
00250 Alanine, aspartate and glutamate metabolism [PATH:ko00250]
00260 Glycine, serine and threonine metabolism [PATH:ko00260]
00270 Cysteine and methionine metabolism [PATH:ko00270]
00280 Valine, leucine and isoleucine degradation [PATH:ko00280]
00290 Valine, leucine and isoleucine biosynthesis [PATH:ko00290]
00300 Lysine biosynthesis [PATH:ko00300]
00310 Lysine degradation [PATH:ko00310]
00311 Penicillin and cephalosporin biosynthesis [PATH:ko00311]
00330 Arginine and proline metabolism [PATH:ko00330]
00340 Histidine metabolism [PATH:ko00340]
00350 Tyrosine metabolism [PATH:ko00350]
00361 gamma−Hexachlorocyclohexane degradation [PATH:ko00361]
00362 Benzoate degradation via hydroxylation [PATH:ko00362]
00380 Tryptophan metabolism [PATH:ko00380]
00400 Phenylalanine, tyrosine and tryptophan biosynthesis [PATH:ko00400]
00430 Taurine and hypotaurine metabolism [PATH:ko00430]
00450 Selenoamino acid metabolism [PATH:ko00450]
00460 Cyanoamino acid metabolism [PATH:ko00460]
00471 D−Glutamine and D−glutamate metabolism [PATH:ko00471]
00473 D−Alanine metabolism [PATH:ko00473]
00480 Glutathione metabolism [PATH:ko00480]
00500 Starch and sucrose metabolism [PATH:ko00500]
00510 N−Glycan biosynthesis [PATH:ko00510]
00511 Other glycan degradation [PATH:ko00511]
00520 Amino sugar and nucleotide sugar metabolism [PATH:ko00520]
00540 Lipopolysaccharide biosynthesis [PATH:ko00540]
00550 Peptidoglycan biosynthesis [PATH:ko00550]
00561 Glycerolipid metabolism [PATH:ko00561]
00562 Inositol phosphate metabolism [PATH:ko00562]
00564 Glycerophospholipid metabolism [PATH:ko00564]
00600 Sphingolipid metabolism [PATH:ko00600]
00601 Glycosphingolipid biosynthesis − lacto and neolacto series [PATH:ko00601]
00603 Glycosphingolipid biosynthesis − globo series [PATH:ko00603]
00620 Pyruvate metabolism [PATH:ko00620]
00624 1− and 2−Methylnaphthalene degradation [PATH:ko00624]
00626 Naphthalene and anthracene degradation [PATH:ko00626]
00628 Fluorene degradation [PATH:ko00628]
00630 Glyoxylate and dicarboxylate metabolism [PATH:ko00630]
00632 Benzoate degradation via CoA ligation [PATH:ko00632]
00640 Propanoate metabolism [PATH:ko00640]
00641 3−Chloroacrylic acid degradation [PATH:ko00641]
00642 Ethylbenzene degradation [PATH:ko00642]
00650 Butanoate metabolism [PATH:ko00650]
00660 C5−Branched dibasic acid metabolism [PATH:ko00660]
00670 One carbon pool by folate [PATH:ko00670]
00680 Methane metabolism [PATH:ko00680]
00710 Carbon fixation in photosynthetic organisms [PATH:ko00710]
00720 Reductive carboxylate cycle (CO2 fixation) [PATH:ko00720]
00730 Thiamine metabolism [PATH:ko00730]
00740 Riboflavin metabolism [PATH:ko00740]
00750 Vitamin B6 metabolism [PATH:ko00750]
00760 Nicotinate and nicotinamide metabolism [PATH:ko00760]
00770 Pantothenate and CoA biosynthesis [PATH:ko00770]
00790 Folate biosynthesis [PATH:ko00790]
00830 Retinol metabolism [PATH:ko00830]
00860 Porphyrin and chlorophyll metabolism [PATH:ko00860]
00900 Terpenoid backbone biosynthesis [PATH:ko00900]
00903 Limonene and pinene degradation [PATH:ko00903]
00908 Zeatin biosynthesis [PATH:ko00908]
00910 Nitrogen metabolism [PATH:ko00910]
00970 Aminoacyl−tRNA biosynthesis [PATH:ko00970]
00980 Metabolism of xenobiotics by cytochrome P450 [PATH:ko00980]
00982 Drug metabolism − cytochrome P450 [PATH:ko00982]
00983 Drug metabolism − other enzymes [PATH:ko00983]
01001 Protein kinases [BR:ko01001]
01002 Peptidases [BR:ko01002]
01003 Glycosyltransferases [BR:ko01003]
01004 Lipid biosynthesis proteins [BR:ko01004]
01005 Lipopolysaccharide biosynthesis proteins [BR:ko01005]
02000 Transporters [BR:ko02000]
02010 ABC transporters [PATH:ko02010]
02020 Two−component system [PATH:ko02020]
02022 Two−component system [BR:ko02022]
02042 Bacterial toxins [BR:ko02042]
02044 Secretion system [BR:ko02044]
02060 Phosphotransferase system (PTS) [PATH:ko02060]
03000 Transcription factors [BR:ko03000]
03010 Ribosome [PATH:ko03010]
03011 Ribosome [BR:ko03011]
03012 Translation factors [BR:ko03012]
03018 RNA degradation [PATH:ko03018]
03020 RNA polymerase [PATH:ko03020]
03030 DNA replication [PATH:ko03030]
03032 DNA replication proteins [BR:ko03032]
03036 Chromosome [BR:ko03036]
03060 Protein export [PATH:ko03060]
03070 Bacterial secretion system [PATH:ko03070]
03110 Chaperones and folding catalysts [BR:ko03110]
03400 DNA repair and recombination proteins [BR:ko03400]
03410 Base excision repair [PATH:ko03410]
03420 Nucleotide excision repair [PATH:ko03420]
03430 Mismatch repair [PATH:ko03430]
03440 Homologous recombination [PATH:ko03440]
04070 Phosphatidylinositol signaling system [PATH:ko04070]
04112 Cell cycle − Caulobacter [PATH:ko04112]
04146 Peroxisome [PATH:ko04146]
04626 Plant−pathogen interaction [PATH:ko04626]
04812 Cytoskeleton proteins [BR:ko04812]
04930 Type II diabetes mellitus [PATH:ko04930]
05010 Alzheimer's disease [PATH:ko05010]
05111 Vibrio cholerae pathogenic cycle [PATH:ko05111]
05340 Primary immunodeficiency [PATH:ko05340]
Glycolysis / Gluconeogenesis [PATH:ko00010]

Figure S8B

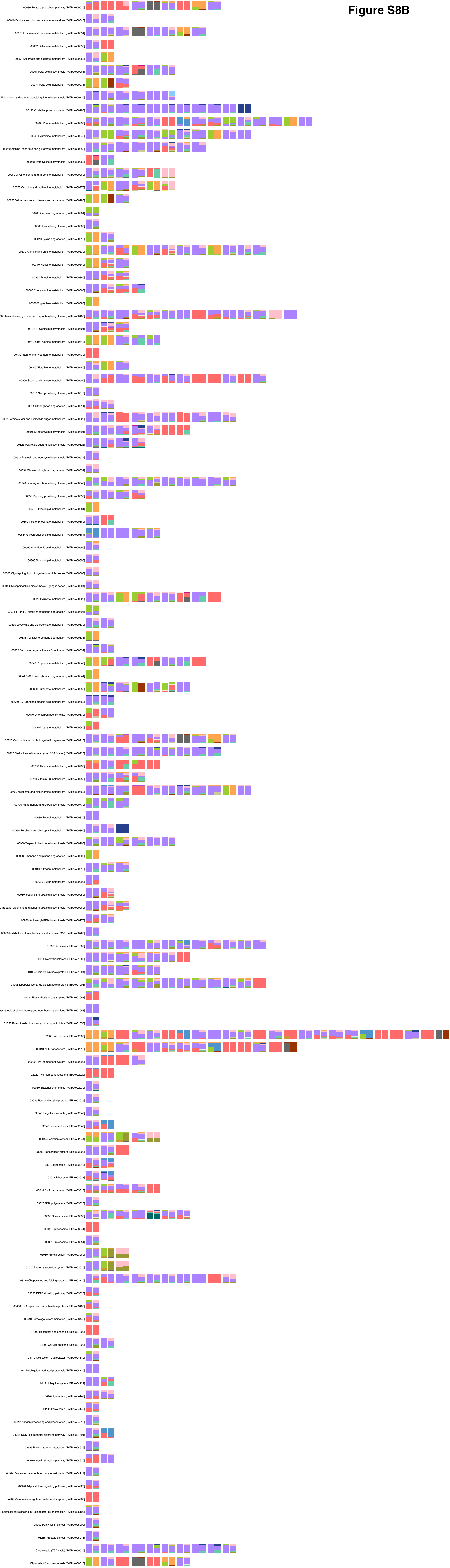

00030 Pentose phosphate pathway [PATH:ko00030]
00040 Pentose and glucuronate interconversions [PATH:ko00040]
00051 Fructose and mannose metabolism [PATH:ko00051]
00052 Galactose metabolism [PATH:ko00052]
00053 Ascorbate and aldarate metabolism [PATH:ko00053]
00061 Fatty acid biosynthesis [PATH:ko00061]
00071 Fatty acid metabolism [PATH:ko00071]
Ubiquinone and other terpenoid−quinone biosynthesis [PATH:ko00130]
00190 Oxidative phosphorylation [PATH:ko00190]
00230 Purine metabolism [PATH:ko00230]
00240 Pyrimidine metabolism [PATH:ko00240]
00250 Alanine, aspartate and glutamate metabolism [PATH:ko00250]
00253 Tetracycline biosynthesis [PATH:ko00253]
00260 Glycine, serine and threonine metabolism [PATH:ko00260]
00270 Cysteine and methionine metabolism [PATH:ko00270]
00280 Valine, leucine and isoleucine degradation [PATH:ko00280]
00281 Geraniol degradation [PATH:ko00281]
00300 Lysine biosynthesis [PATH:ko00300]
00310 Lysine degradation [PATH:ko00310]
00330 Arginine and proline metabolism [PATH:ko00330]
00340 Histidine metabolism [PATH:ko00340]
00350 Tyrosine metabolism [PATH:ko00350]
00360 Phenylalanine metabolism [PATH:ko00360]
00380 Tryptophan metabolism [PATH:ko00380]
0 Phenylalanine, tyrosine and tryptophan biosynthesis [PATH:ko00400]
00401 Novobiocin biosynthesis [PATH:ko00401]
00410 beta−Alanine metabolism [PATH:ko00410]
00430 Taurine and hypotaurine metabolism [PATH:ko00430]
00480 Glutathione metabolism [PATH:ko00480]
00500 Starch and sucrose metabolism [PATH:ko00500]
00510 N−Glycan biosynthesis [PATH:ko00510]
00511 Other glycan degradation [PATH:ko00511]
00520 Amino sugar and nucleotide sugar metabolism [PATH:ko00520]
00521 Streptomycin biosynthesis [PATH:ko00521]
00523 Polyketide sugar unit biosynthesis [PATH:ko00523]
00524 Butirosin and neomycin biosynthesis [PATH:ko00524]
00531 Glycosaminoglycan degradation [PATH:ko00531]
00540 Lipopolysaccharide biosynthesis [PATH:ko00540]
00550 Peptidoglycan biosynthesis [PATH:ko00550]
00561 Glycerolipid metabolism [PATH:ko00561]
00562 Inositol phosphate metabolism [PATH:ko00562]
00564 Glycerophospholipid metabolism [PATH:ko00564]
00590 Arachidonic acid metabolism [PATH:ko00590]
00600 Sphingolipid metabolism [PATH:ko00600]
00603 Glycosphingolipid biosynthesis − globo series [PATH:ko00603]
00604 Glycosphingolipid biosynthesis − ganglio series [PATH:ko00604]
00620 Pyruvate metabolism [PATH:ko00620]
00624 1− and 2−Methylnaphthalene degradation [PATH:ko00624]
00630 Glyoxylate and dicarboxylate metabolism [PATH:ko00630]
00631 1,2−Dichloroethane degradation [PATH:ko00631]
00632 Benzoate degradation via CoA ligation [PATH:ko00632]
00640 Propanoate metabolism [PATH:ko00640]
00641 3−Chloroacrylic acid degradation [PATH:ko00641]
00650 Butanoate metabolism [PATH:ko00650]
00660 C5−Branched dibasic acid metabolism [PATH:ko00660]
00670 One carbon pool by folate [PATH:ko00670]
00680 Methane metabolism [PATH:ko00680]
00710 Carbon fixation in photosynthetic organisms [PATH:ko00710]
00720 Reductive carboxylate cycle (CO2 fixation) [PATH:ko00720]
00730 Thiamine metabolism [PATH:ko00730]
00750 Vitamin B6 metabolism [PATH:ko00750]
00760 Nicotinate and nicotinamide metabolism [PATH:ko00760]
00770 Pantothenate and CoA biosynthesis [PATH:ko00770]
00830 Retinol metabolism [PATH:ko00830]
00860 Porphyrin and chlorophyll metabolism [PATH:ko00860]
00900 Terpenoid backbone biosynthesis [PATH:ko00900]
00903 Limonene and pinene degradation [PATH:ko00903]
00910 Nitrogen metabolism [PATH:ko00910]
00920 Sulfur metabolism [PATH:ko00920]
00950 Isoquinoline alkaloid biosynthesis [PATH:ko00950]
) Tropane, piperidine and pyridine alkaloid biosynthesis [PATH:ko00960]
00970 Aminoacyl−tRNA biosynthesis [PATH:ko00970]
00980 Metabolism of xenobiotics by cytochrome P450 [PATH:ko00980]
01002 Peptidases [BR:ko01002]
01003 Glycosyltransferases [BR:ko01003]
01004 Lipid biosynthesis proteins [BR:ko01004]
01005 Lipopolysaccharide biosynthesis proteins [BR:ko01005]
01051 Biosynthesis of ansamycins [PATH:ko01051]
ynthesis of siderophore group nonribosomal peptides [PATH:ko01053]
01055 Biosynthesis of vancomycin group antibiotics [PATH:ko01055]
02000 Transporters [BR:ko02000]
02010 ABC transporters [PATH:ko02010]
02020 Two−component system [PATH:ko02020]
02022 Two−component system [BR:ko02022]
02030 Bacterial chemotaxis [PATH:ko02030]
02035 Bacterial motility proteins [BR:ko02035]
02040 Flagellar assembly [PATH:ko02040]
02042 Bacterial toxins [BR:ko02042]
02044 Secretion system [BR:ko02044]
03000 Transcription factors [BR:ko03000]
03010 Ribosome [PATH:ko03010]
03011 Ribosome [BR:ko03011]
03018 RNA degradation [PATH:ko03018]
03020 RNA polymerase [PATH:ko03020]
03036 Chromosome [BR:ko03036]
03041 Spliceosome [BR:ko03041]
03051 Proteasome [BR:ko03051]
03060 Protein export [PATH:ko03060]
03070 Bacterial secretion system [PATH:ko03070]
03110 Chaperones and folding catalysts [BR:ko03110]
03320 PPAR signaling pathway [PATH:ko03320]
03400 DNA repair and recombination proteins [BR:ko03400]
03440 Homologous recombination [PATH:ko03440]
04000 Receptors and channels [BR:ko04000]
04090 Cellular antigens [BR:ko04090]
04112 Cell cycle − Caulobacter [PATH:ko04112]
04120 Ubiquitin mediated proteolysis [PATH:ko04120]
04121 Ubiquitin system [BR:ko04121]
04142 Lysosome [PATH:ko04142]
04146 Peroxisome [PATH:ko04146]
04612 Antigen processing and presentation [PATH:ko04612]
04621 NOD−like receptor signaling pathway [PATH:ko04621]
04626 Plant−pathogen interaction [PATH:ko04626]
04910 Insulin signaling pathway [PATH:ko04910]
04914 Progesterone−mediated oocyte maturation [PATH:ko04914]
04920 Adipocytokine signaling pathway [PATH:ko04920]
04962 Vasopressin−regulated water reabsorption [PATH:ko04962]
) Epithelial cell signaling in Helicobacter pylori infection [PATH:ko05120]
05200 Pathways in cancer [PATH:ko05200]
05215 Prostate cancer [PATH:ko05215]
Citrate cycle (TCA cycle) [PATH:ko00020]
Glycolysis / Gluconeogenesis [PATH:ko00010]

Figure S9

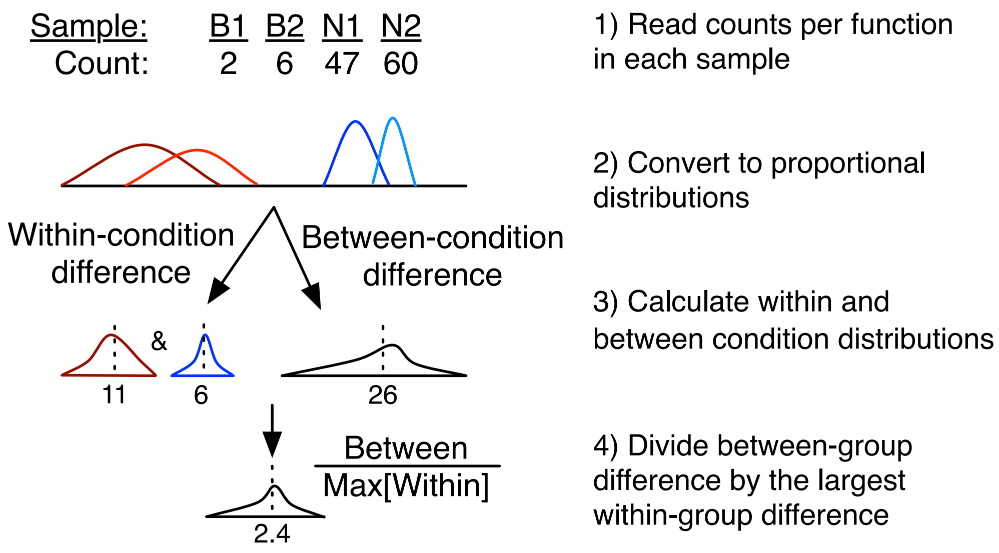

Sample:
B1
B2
N1
N2
Count:
2
6
47
60
1) Read counts per function in each sample
2) Convert to proportional distributions
Within-condition difference
Between-condition difference
3) Calculate within and between condition distributions
&
11
6
26
Between
Max[Within]
4) Divide between-group difference by the largest within-group difference
2.4

Figure S10

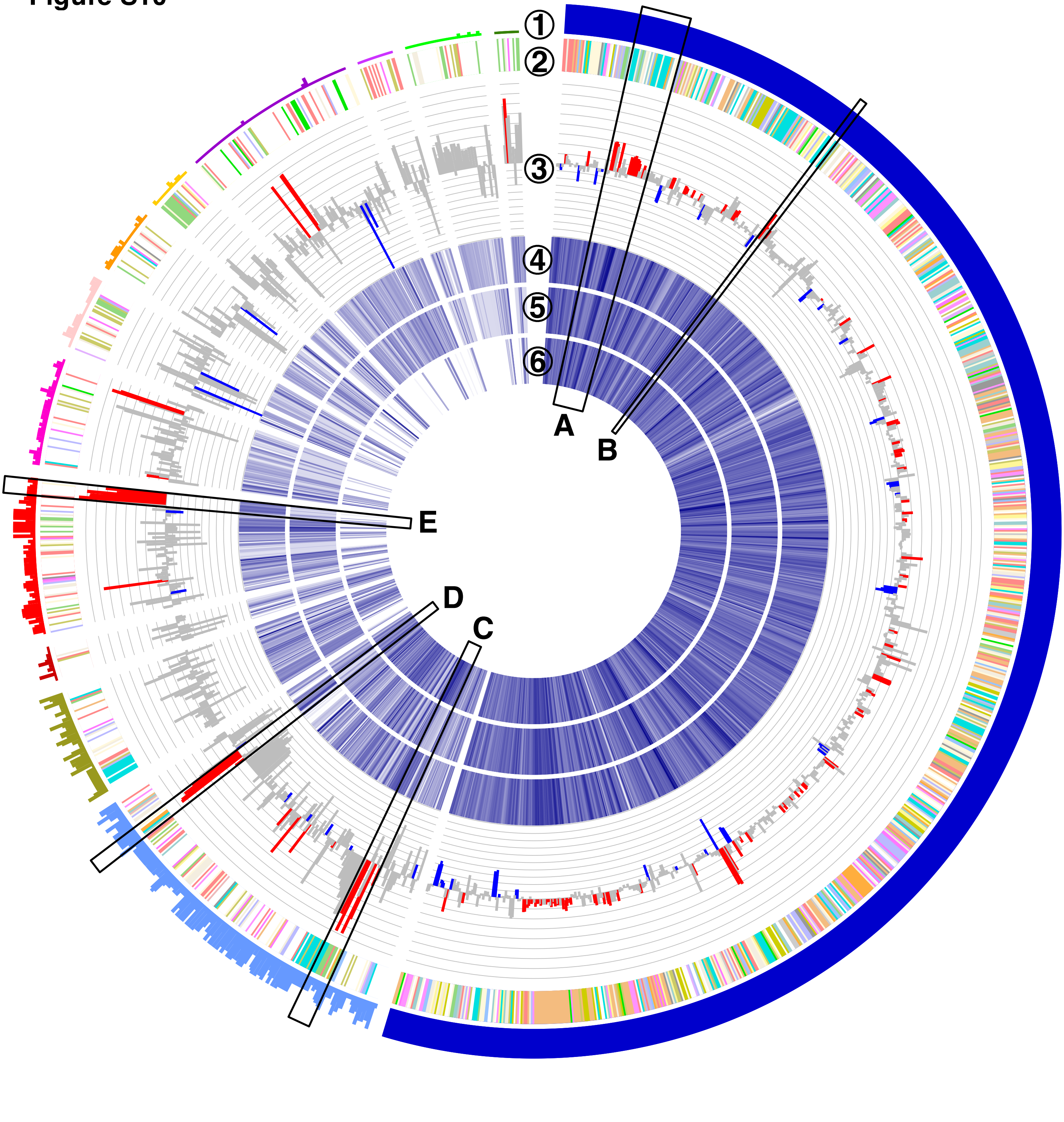

Table S1. Nugent scoring results

Sample ID	Field No.	Lactobacillus	Score	Gardnerella	Score	Mobiluncus	Score	Total Score	pH
	1	>30		0		0			
	2	>30		4		0			
**N4**	3	>30		2		0		**2**	<= 4.5
	4	>30		4		0			
	**Average**	**>30**	**0**	**2.5**	**2**	**0**	**0**		
	1	>30		1		0			
	2	>30		6		0			
**N30**	3	>30		0		0		**2**	<= 4.5
	4	>30		2		0			
	**Average**	**>30**	**0**	**2.25**	**2**	**0**	**0**		
	1	0		>30		>30			
	2	0		>30		>30			
**B27**	3	0		>30		>30		**10**	5.5
	4	0		>30		>30			
	**Average**	**0**	**4**	**>30**	**4**	**>30**	**2**		
	1	16		>30		>30			
	2	12		>30		>30			
**B31**	3	12		>30		>30		**7**	5.0
	4	3		>30		>30			
	**Average**	**10.75**	**1**	**>30**	**4**	**>30**	**2**		

Table S2. Sequence and mapping data.

		N4	N30	B27	B31	Total
**Raw reads**		47,634,967	46,943,184	48,832,687	53,655,422	197,066,260
**Reads mapped to human genome (excluded)**		2,649,739<br>5.56%	4,525,289<br>9.64%	1,385,096<br>2.84%	300,792<br>0.56%	8,860,916<br>4.50%
**Reads mapped to bacterial library**	**Total mapped**	**20,556,493**	**14,690,153**	**11,981,948**	**12,556,674**	**59,785,268**
	Unique	12,178,729	10,260,422	9,321,042	8,349,061	40,109,254
	Non-unique	8,377,764	4,429,731	2,660,906	4,207,613	19,676,014
	**Unique mapped to CDS***	**10,635,713**	**8,809,565**	**7,469,729**	**5,487,128**	**32,402,135**
**No. of unique refseqs mapped**		10,770	11,745	20,162	22,860	**33,412**†
**No. refseqs assigned to KEGG**	18507 (55.4%)	2056 unique KOs				
**No. refseqs assigned to SEED subsys4**	22735 (68.0%)	2772 unique subsys4				
**No.refseqs assigned to COG**	25951 (77.7%)	2300 unique COGs				

*These are reads mapping uniquely to coding sequences (CDS). These reads were used for differential expression analysis.

†This is the total number of unique reference sequence CDS (refseqs) mapped across all samples

Table S3. Read counts after mapping to curtated, non-redundant cpn60 database

**Taxon**	**4N**	**30N**	**27B**	**31B**
Lactobacillus_iners	1060	6910	2759	5284
Lactobacillus_crispatus	14261	7010	0	707
Gardnerella_vaginalis	0	0	408	308
Megasphaera_sp	0	0	3202	0
Prevotella_amnii	15	12	34186	3113
Prevotella_disiens	0	0	0	918
Prevotella_timonensis	0	0	220	688
Lactobacillus_jensenii	222	194	0	17
Lactobacillus_johnsonii	22	0	0	0
Lactobacillus_kefironofacieus	190	0	0	0
Lactobacillus_rhamnosus	17	13	18	20
Lactobacillus_acidophilus	14	12	0	0
Lactobacillus_ruminis	0	0	46	0
Alloscardovia_omnicolens	0	0	15	0
Atopobium_vaginae	0	0	50	12
Clostridium_genomosp_BVAB3	0	0	33	27
Fusobacterium_sp	0	0	23	0
Peptoniphilus_lacrimalis	0	0	0	48
Porphyromonas_uenonis	0	0	37	0
Prevotella_bivia	0	0	21	0
Prevotella_buccalis	0	0	42	14
Prevotella_melaninogenica	0	0	0	30
Streptococcus_thermophilus	145	102	81	94
Vagococcus_fluvialis	49	0	30	54
**total_reads**	15995	14253	41171	11334

Table S4. List of organisms used to build reference mapping library

Organism name	GenBank or Refseq accession	No. of CDS
Actinomyces coleocanis DSM 15436	ACFG00000000	1546
Actinomyces urogenitalis DSM 15434	ACFH00000000	2403
Aerococcus viridans ATCC 11563	ADNT00000000	1929
Anaerococcus lactolyticus ATCC 51172	ABYO00000000	2253
Anaerococcus tetradius ATCC 35098	ACGC00000000	2079
Atopobium vaginae DSM 15829	ACGK00000000	1271
Atopobium vaginae PB189-T1-4	AEDQ00000000	1217
Bifidobacterium dentium ATCC 27679	AEEQ00000000	2336
Bifidobacterium dentium JCVIHMP022	AEHJ00000000	2278
Brevibacterium mcbrellneri ATCC 49030	ADNU00000000	2432
Chryseobacterium gleum F93, ATCC 35910	ACKQ00000000	5289
Clostridiales genomosp. BVAB3 UPII9-5	CP001850	1526
Corynebacterium aurimucosum ATCC 700975	ACLH00000000	2614
Corynebacterium genitalium ATCC 33030	ACLJ00000000	2226
Corynebacterium glucuronalyticum ATCC 51867	ABYP00000000	2645
Corynebacterium glucuronolyticum ATCC 51866	ACHF00000000	2733
Corynebacterium jeikeium ATCC 43734	ACYW00000000	2224
Corynebacterium lipophiloflavum DSM 44291	ACHJ00000000	2371
Corynebacterium pseudogenitalium ATCC 33035	ABYQ00000000	2607
Corynebacterium striatum ATCC 6940	ACGE00000000	2677
Dialister microaerophilus UPII-345-E	AENT00000000	1310
Enterococcus faecalis ATCC 29200	ACHK00000000	2955
Enterococcus faecalis HH22	ACIX00000000	3227
Enterococcus faecalis TX 0312	AECB00000000	2789
Enterococcus faecalis TX 0635	AEBZ00000000	3263
Enterococcus faecalis TX0855	AEBV00000000	3032
Eremococcus coleocola ACS-139-V-Col8	AENN00000000	1720
Escherichia coli 83972	ACGN00000000	5255
Finegoldia magna ACS-171-V-Col3	AECM00000000	1740
Finegoldia magna ATCC 29328	NC_010371	182
Finegoldia magna ATCC 29328	NC_010376	1631
Finegoldia magna ATCC 53516	ACHM00000000	1838
Finegoldia magna BVS033A4	AEDP00000000	1904
Fusobacterium nucleatum nucleatum ATCC 23726	ADVK00000000	2126
Gardnerella vaginalis ATCC 14019	NC_014644	1365
Gardnerella vaginalis 409-05	NC_013721	1261
Gardnerella vaginalis AMD	ADAM00000000	1313
Lactobacillus acidophilus NCFM	NC_006814	1864
Lactobacillus crispatus 214-1	ADGR00000000	2163
Lactobacillus crispatus JV-V01	ACKR00000000	2209
Lactobacillus crispatus MV-3A-US	ACQC00000000	2330
Lactobacillus crispatus ST1	NC_014106	2024
Lactobacillus delbrueckii bulgaricus PB2003/044-T3-4	AEAT00000000	1909
Lactobacillus fermentum IFO	NC_010610	1843
Lactobacillus gasseri 202-4	ACOZ00000000	1773
Lactobacillus gasseri 224-1	ADFT00000000	2252
Lactobacillus gasseri JV-V03	ACGO00000000	1977
Lactobacillus gasseri MV-22	ABWH00000000	1917
Lactobacillus iners AB-1	ADHG00000000	1190
Lactobacillus iners ATCC 55195	AEPX00000000	1144
Lactobacillus iners DSM 13335	ACLN00000000	1214
Lactobacillus iners LactinV 01V1-a	AEHQ00000000	1527
Lactobacillus iners LactinV 03V1-b	AEHP00000000	1459
Lactobacillus iners LactinV 09V1-c	AEHO00000000	1361
Lactobacillus iners LactinV 11V1-d	AEHN00000000	1338

Organism name	GenBank or Refseq accession	No. of CDS
Lactobacillus iners LEAF 2052A-d	AEKI00000000	1256
Lactobacillus iners LEAF 2053A-b	AEKH00000000	1277
Lactobacillus iners LEAF 2062A-h1	AEKJ00000000	1265
Lactobacillus iners LEAF 3008A-a	AEKK00000000	1210
Lactobacillus iners SPIN 2503V10-D	AEHR00000000	1273
Lactobacillus jensenii 1153	ABWG00000000	1395
Lactobacillus jensenii 208-1	ADEX00000000	3182
Lactobacillus jensenii 269-3	ACOY00000000	1575
Lactobacillus jensenii JV-V16	ACGQ00000000	1450
Lactobacillus johnsonii ATCC 33200	ACGR00000000	1838
Lactobacillus johnsonii FI9785	NC_012552	2
Lactobacillus johnsonii FI9785	NC_013504	1710
Lactobacillus johnsonii FI9785	NC_013505	23
Lactobacillus johnsonii FI9785	NC_005362	1821
Lactobacillus oris PB013-T2-3	AEKL00000000	2038
Lactobacillus reuteri_JCM 1112	NC_010609	1820
Lactobacillus rhamnosus_Lc 705	NC_013199	2878
Lactobacillus rhamnosus_Lc 705	NC_013200	79
Lactobacillus salivarius ACS-116-V-Col5a	AEBA00000000	2121
Lactobacillus vaginalis ATCC 49540	ACGV00000000	1870
Leptotrichia buccalis C 1013	NC_013192	2220
Megasphaera genomosp. type_1 28L	ADGP00000000	1610
Mobiluncus curtisii ATCC 43063	NC_014246	1909
Mobiluncus curtisii ATCC 51333	AEPY00000000	1855
Mobiluncus curtisii curtisii ATCC 35241	AEEE00000000	1894
Mobiluncus curtisii holmesii ATCC 35242	AEPZ00000000	1829
Mobiluncus mulieris 28-1	ADBR00000000	2321
Mobiluncus mulieris ATCC 35239	AEET00000000	2380
Mobiluncus mulieris ATCC 35243	ACKW00000000	2300
Mobiluncus mulieris FB024-16	AEGV00000000	2113
Mycobacterium parascrofulaceum ATCC BAA-614	ADNV00000000	6456
Peptoniphilus duerdenii ATCC BAA-1640	AEEH00000000	1988
Peptoniphilus lacrimalis 315-B	ADDO00000000	1589
Porphyromonas asaccharolytica PR426713P-I	AENO00000000	1655
Porphyromonas uenonis 60-3	ACLR00000000	1977
Prevotella amnii CRIS 21A-A	ADFQ00000000	2025
Prevotella bivia JCVIHMP010	ADFO00000000	2041
Prevotella buccalis ATCC 35310	ADEG00000000	2456
Prevotella disiens FB035-09AN	AEDO00000000	2621
Prevotella melaninogenica ATCC 25845	NC_014370	1338
Prevotella melaninogenica ATCC 25845	NC_014371	958
Prevotella oralis ATCC 33269	AEPE00000000	2488
Prevotella timonensis CRIS 5C-B1	ADEF00000000	2202
Proteus mirabilis ATCC 29906	ACLE00000000	3812
Roseomonas cervicalis ATCC 49957	ADVL00000000	4778
Sphingobacterium spiritivorum ATCC 33300	ACHB00000000	4925
Sphingobacterium spiritivorum ATCC 33861	ACHA00000000	4471
Staphylococcus aureus aureus MN8	ACJA00000000	2833
Staphylococcus aureus COL	NC_002951	2612
Staphylococcus aureus COL	NC_006629	3
Streptococcus agalactiae 2603V	NC_004116	2124
Streptococcus bovis ATCC 700338	AEEL00000000	2088
Streptococcus pseudoporcinus SPIN 20026	AENS00000000	2030
Treponema phagedenis F0421	AEFH00000000	3146
Veillonella atypica ACS-049-V-Sch6	AEDR00000000	1840
**Total**		**230031**

Table S5. Read coverage of *Lactobacillus iners* coding sequences (CDS)

**Average number of CDS***	1293
**Average CDS length (nt)**	970
Product	**1254210**

	**N4**	**N30**	**N27**	**N31**
**Total mapped reads to L. iners' CDS**	122332	617960	1928856	2931047
**x 50 bp per read**	6116600	30898000	96442800	146552350
**Approx. CDS coverage**	**4.88**	**24.64**	**76.90**	**116.85**

*See Table S4

Table S6. Summary of reads mapped to CRISPR spacer sequences

CRISPR spacer sequence ID		N4		N30		B27		B31	
		**No. reads**	**% of total mapped reads**	**No. reads**	**% of total mapped reads**	**No. reads**	**% of total mapped reads**	**No. reads**	**% of total mapped reads**
lcl|1713|ref|NC_007716|	Aster yellows witches'-broom phytoplasma AYWB 277436-277525	0	0.00	0	0.00	0	0.00	1	0.00
lcl|1714|ref|NC_007716|	Aster yellows witches'-broom phytoplasma AYWB 277342-277417	58	0.03	109	0.07	15	0.07	55	0.06
lcl|1716|ref|NC_007716|	Aster yellows witches'-broom phytoplasma AYWB 277167-277226	0	0.00	0	0.00	1	0.00	0	0.00
lcl|2498|ref|NC_011891|	Anaeromyxobacter dehalogenans 2CP-1 3151161-3151315	0	0.00	1	0.00	0	0.00	0	0.00
lcl|3269|ref|NC_009674|	Bacillus cereus subsp. cytotoxis NVH 391-98 3571402-3571478	8	0.00	18	0.01	3	0.01	7	0.01
lcl|3270|ref|NC_009674|	Bacillus cereus subsp. cytotoxis NVH 391-98 3571303-3571377	10	0.00	95	0.06	228	1.06	779	0.81
lcl|3272|ref|NC_009674|	Bacillus cereus subsp. cytotoxis NVH 391-98 3571141-3571198	21	0.01	250	0.16	95	0.44	356	0.37
lcl|10652|ref|NC_008593|	Clostridium novyi NT 1866066-1866100	2	0.00	0	0.00	0	0.00	0	0.00
lcl|13253|ref|NC_012785|	Kosmotoga olearia TBF 19.5.1 723753-723786	0	0.00	0	0.00	0	0.00	1	0.00
lcl|13330|ref|NC_010080|	Lactobacillus helveticus DPC 4571 1587946-1587980	15	0.01	17	0.01	0	0.00	0	0.00
lcl|13337|ref|NC_010080|	Lactobacillus helveticus DPC 4571 1695949-1696043	101829	45.21	63058	41.28	14304	66.60	56128	58.03
lcl|13338|ref|NC_010080|	Lactobacillus helveticus DPC 4571 1695809-1695937	90804	40.31	63592	41.63	6194	28.84	31881	32.96
lcl|13339|ref|NC_010080|	Lactobacillus helveticus DPC 4571 1695675-1695797	27975	12.42	24092	15.77	15	0.07	5400	5.58
lcl|13340|ref|NC_010080|	Lactobacillus helveticus DPC 4571 1695542-1695663	2698	1.20	1254	0.82	441	2.05	1408	1.46
lcl|15149|ref|NC_006360|	Mycoplasma hyopneumoniae 232 338057-338117	0	0.00	0	0.00	45	0.21	33	0.03
lcl|15151|ref|NC_006360|	Mycoplasma hyopneumoniae 232 337855-337962	1	0.00	1	0.00	22	0.10	178	0.18
lcl|16408|ref|NC_013316|	Clostridium difficile R20291 chromosome 1292772-1292809	1	0.00	8	0.01	0	0.00	0	0.00
lcl|16502|ref|NC_010376|	Finegoldia magna ATCC 29328 chromosome 197417-197654	0	0.00	0	0.00	0	0.00	3	0.00
lcl|16505|ref|NC_010376|	Finegoldia magna ATCC 29328 chromosome 196644-196855	0	0.00	0	0.00	0	0.00	4	0.00
lcl|17873|ref|NC_009454|	Pelotomaculum thermopropionicum SI chromosome 1998702-1998738	0	0.00	0	0.00	1	0.00	0	0.00
lcl|18404|ref|NC_006814|	Lactobacillus acidophilus NCFM chromosome 1541067-1541098	13	0.01	0	0.00	0	0.00	0	0.00
lcl|18406|ref|NC_006814|	Lactobacillus acidophilus NCFM chromosome 1542898-1542929	37	0.02	30	0.02	33	0.15	122	0.13
lcl|20031|ref|NC_012032|	Chloroflexus sp. Y-400-fl chromosome 4548942-4548978	0	0.00	0	0.00	8	0.04	17	0.02
lcl|20808|ref|NC_013511|	Mycoplasma hominis 259090-259162	0	0.00	0	0.00	0	0.00	1	0.00
lcl|25524|ref|NC_009523|	Roseiflexus sp. RS-1 chromosome 3157153-3157189	0	0.00	3	0.00	0	0.00	1	0.00
lcl|25767|ref|NC_014752|	Neisseria lactamica ST-640 1672927-1672964	1770	0.79	229	0.15	66	0.31	310	0.32
lcl|26144|ref|NC_014484|	Spirochaeta thermophila DSM 6192 chromosome 461133-461170	0	0.00	0	0.00	1	0.00	0	0.00
lcl|31970|ref|NC_000919|	Treponema pallidum subsp. pallidum str. Nichols chromosome 77273-77566	0	0.00	0	0.00	0	0.00	15	0.02
lcl|32709|ref|NC_010175|	Chloroflexus aurantiacus J-10-fl chromosome 4538893-4538930	0	0.00	0	0.00	5	0.02	17	0.02
lcl|33777|ref|NC_010741|	Treponema pallidum subsp. pallidum SS14 77270-77563	0	0.00	0	0.00	0	0.00	10	0.01
lcl|39861|ref|NC_015499|	Thermodesulfobium narugense DSM 14796 chromosome 1770147-1770185	0	0.00	0	0.00	0	0.00	1	0.00
lcl|35999|ref|NC_013715|	Rothia mucilaginosa DY-18 chromosome 1380875-1381203	0	0.00	1	0.00	0	0.00	0	0.00
lcl|40601|ref|NC_009437|	Caldicellulosiruptor saccharolyticus DSM 8903 chromosome 357323-357358	0	0.00	0	0.00	0	0.00	2	0.00
**Total reads mapped**		**225242**		**152758**		**21477**		**96730**	
